# *Pseudomonas aeruginosa*: pathogenesis, virulence factors, antibiotic resistance, interaction with host, technology advances and emerging therapeutics

**DOI:** 10.1038/s41392-022-01056-1

**Published:** 2022-06-25

**Authors:** Shugang Qin, Wen Xiao, Chuanmin Zhou, Qinqin Pu, Xin Deng, Lefu Lan, Haihua Liang, Xiangrong Song, Min Wu

**Affiliations:** 1 Department of Critical Care Medicine, Frontiers Science Center for Disease-related Molecular Network, State Key Laboratory of Biotherapy and Cancer Center, West China Hospital, Sichuan University, Chengdu, China; 2 State Key Laboratory of Virology, School of Public Health, Wuhan University, Wuhan, 430071 P.R. China; 3 Department of Biomedical Sciences, School of Medicine and Health Sciences, University of North Dakota, Grand Forks, ND 58203 USA; 4 Department of Biomedical Sciences, City University of Hong Kong, Hong Kong, People’s Republic of China; 5 State Key Laboratory of Drug Research, Shanghai Institute of Materia Medica, Chinese Academy of Sciences, Shanghai, 201203 China; 6 College of Life Sciences, Northwest University, Xi’an, ShaanXi 710069 China

**Keywords:** Microbiology, Infection

## Abstract

*Pseudomonas aeruginosa* (*P. aeruginosa*) is a Gram-negative opportunistic pathogen that infects patients with cystic fibrosis, burn wounds, immunodeficiency, chronic obstructive pulmonary disorder (COPD), cancer, and severe infection requiring ventilation, such as COVID-19. *P. aeruginosa* is also a widely-used model bacterium for all biological areas. In addition to continued, intense efforts in understanding bacterial pathogenesis of *P. aeruginosa* including virulence factors (LPS, quorum sensing, two-component systems, 6 type secretion systems, outer membrane vesicles (OMVs), CRISPR-Cas and their regulation), rapid progress has been made in further studying host-pathogen interaction, particularly host immune networks involving autophagy, inflammasome, non-coding RNAs, cGAS, *etc*. Furthermore, numerous technologic advances, such as bioinformatics, metabolomics, scRNA-seq, nanoparticles, drug screening, and phage therapy, have been used to improve our understanding of *P. aeruginosa* pathogenesis and host defense. Nevertheless, much remains to be uncovered about interactions between *P. aeruginosa* and host immune responses, including mechanisms of drug resistance by known or unannotated bacterial virulence factors as well as mammalian cell signaling pathways. The widespread use of antibiotics and the slow development of effective antimicrobials present daunting challenges and necessitate new theoretical and practical platforms to screen and develop mechanism-tested novel drugs to treat intractable infections, especially those caused by multi-drug resistance strains. Benefited from has advancing in research tools and technology, dissecting this pathogen’s feature has entered into molecular and mechanistic details as well as dynamic and holistic views. Herein, we comprehensively review the progress and discuss the current status of *P. aeruginosa* biophysical traits, behaviors, virulence factors, invasive regulators, and host defense patterns against its infection, which point out new directions for future investigation and add to the design of novel and/or alternative therapeutics to combat this clinically significant pathogen.

## Introduction to *Pseudomonas aeruginosa*, a critical clinical pathogen and model microorganism

*Pseudomonas aeruginosa* is a multi-drug resistance (MDR) opportunistic pathogens, causing acute or chronic infection in immunocompromised individuals with chronic obstructive pulmonary disease (COPD), cystic fibrosis, cancer, traumas, burns, sepsis, and ventilator-associated pneumonia (VAP) including those caused by COVID-19.^1–3^ *P. aeruginosa* in biofilm states may survive in a hypoxic atmosphere or other extremely harsh environments.^4,5^ In addition, treatments of *P. aeruginosa* infection are extremely difficult due to its rapid mutations and adaptation to gain resistance to antibiotics.^6^ Furthermore, *P. aeruginosa* is also one of the top-listed pathogens causing hospital-acquired infections, which are widely found in medical devices (ventilation) because they tend to thrive on wet surfaces.^7^ Importantly, *P. aeruginosa* is one of the MDR ESKAPE pathogens, which stand for pathogens *Enterococcus faecium, Staphylococcus aureus, Klebsiella pneumoniae, Acinetobacter baumannii*, *P. aeruginosa*, and *Enterobacter*. *P. aeruginosa* with arbapenem-resistance is listed among the “critical” group of pathogens by WHO, which urgently need novel antibiotics in the clinics.^8^

Epidemiological studies have shown that nearly 700,000 people died of the antibiotic resistance bacterial infections each year. *P. aeruginosa* that was isolated from European populations with a combined resistance was 12.9%.^9^ Hospital-acquired infection caused by *P. aeruginosa* continues to produce resistance to conventionally effective antibiotics becoming a main healthcare problem.^10^ The 2016 EPINE survey found that *Escherichia coli* and *P. aeruginosa* are the most common cause of hospital-acquired infections in Spain, *P. aeruginosa* accounting for 10.5% of clinically isolated bacteria infections.^11^ The 2011–2012 HCAIs report that *P. aeruginosa* caused almost nosocomial 9% of infections, which is the fourth commonest pathogen of the European hospitals.^12^ Similarly, 7.1% of HCAI are caused by *P. aeruginosa* in the United States.^13^ In addition, the 2016 European Center for Disease Prevention and Control (ECDC) epidemiological reported that *P. aeruginosa* causes a variety of ICU-hospital-acquired infections, including pneumonia flares, urinary tract infections, and bloodstream infections.^9,10,14,15^ Furthermore, data from China Antimicrobial Surveillance Network (CHINET) (http://www.chinets.com/) identified 301,917 clinically isolated pathogenic strains and found *P. aeruginosa* was the fourth nosocomial infections after *Escherichia coli*, *Klebsiella pneumoniae*, and *Staphylococcus aureus*, accounting for 7.96%. Altogether, *P. aeruginosa* is not a local, but a global major threat to human health.

**Fig. 1:**
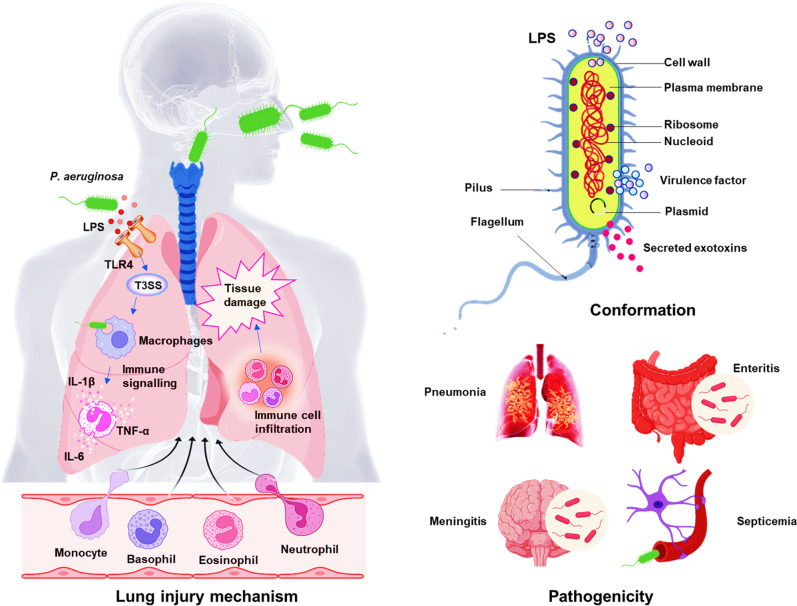
Schema of *P. aeruginosa* pathogenesis. *P. aeruginosa* can be traced everywhere including hospital environments and cause serious infection of almost any organ. LPS induces TLR-4-dependent and -independent inflammatory responses in the lung after bacterial infection, epithelial cells secrete cytokines and chemokines, thereby recruiting and activating innate immune cells and adaptive immune cells. The recruitment of neutrophils is a sign of inflammatory response activation. Although the activation of neutrophils is critical for host defense, excessively activated immune cell infiltration will cause severe tissue damage and aggravate bacterial infections.^478^ Therefore, studying the balance between the virulence factors secreted by bacteria and corresponding host immunity is important for the treatment of infections

The aforementioned statistics necessitate the identification drug targets and development of new treatments and effective vaccines for *P. aeruginosa* to improve human health. However, both efforts have met huge difficulty due to the surging cases with MDR strains. This article broadly reviews the recent progress in *P. aeruginosa* research towards the regulatory and functional mechanisms of virulence factors, gene expression regulators, secretion systems, quorum sensing, and antibiotic resistance, as well as host-pathogen interaction, new technologic advances, and therapeutic development (Fig. 1).

## Virulence factors

*P. aeruginosa* is able to adapt to the adverse environment in hosts by secreting a variety of virulence factors, which contribute to successful infection and causing disease.^16^ First, lipopolysaccharide (LPS) is an important surface structural component to protect the external leaflet and posion host cells and the endotoxicity of the lipid A in LPS enable tissue damage, attachment, and recognition by host receptors.^17^ LPS may be related to antibiotic tolerance and biofilm formation.^18^ Second, out membrane proteins (OMPs) contributes to nutrient exchange, adhesion, and antibiotic resistance.^19^ In addition, drug resistance caused by the formation of biofilms is associated with the flagellum, pili, and other adhesins.^20^ Fourth, there are six types of secretion systems, including flagella (T6SS-associated), pili (T4SS), and multi-toxin components type 3 secretion system (T3SS), which function at colonisation of the host, adhesion, swimming, and swarming responding chemotactic signaling. Finally, exopolysaccharides, such as alginate, Psl and Pel, may help facilitate the formation of biofilms, while impairing bacterial clearance.^20^

In terms of toxins, T3SS is a complex system and may severely impede host defense via injection of cytotoxins including ExoU, ExoT, ExoS and ExoY, which affect the intracellular environment, especially blocking phagocytosis and bacterial clearance. Exotoxin A (ETA) can inhibit host protein synthesis through ADP ribosylation activity.^21,22^ Pyocyanin is also toxic to hosts to cause disease severity, damage host tissue, and impair organs’ function.^23^ In addition, LasA and LasB elastases, alkaline protease (AprA) LipC lipases, phospholipase C, and esterase A enzymes comprise a large group of lytic enzymes that modulate other virulence factors.^24^ Moreover, rhamnolipids-mediated lung surfactant degrading and tight junction destroying can directly injure trachea or lung epithelial cells.^25^ Furthermore, siderophores (pyoverdine and pyochelin) as iron uptake systems help in bacterial survival in iron-depleted environment to augment virulence strength.^26^ Finally, antioxidant enzymes, such as catalases (KatA, KatB, and KatE), alkyl hydroperoxide reductases, and superoxide dismutases, neutralize activity of reactive oxygen species (ROS) in phagocyte environments to avoid clearance.^27^

### Virulence factors related to membranes

#### Lipopolysaccharide as a virulence factor widely interacting with hosts and also target for vaccines

LPS, an important classical structural component of the outer membrane (OM) of most Gram-negative bacteria, is a known potent agonist that elicits robust innate inflammatory immunity, its distal end may be capped with O antigen, a long polysaccharide that can range from a few to hundreds of sugars in length, which is critical for bacterial physiology and pathogenesis.^28^ At the early stage, scientists were interested in developing vaccines to prevent infection by focusing on LPS, which were later proven highly difficult due to the various serotypes and inefficacious outcomes.^28–30^

Pathogen-associated molecular patterns (PAMPs), as small molecular motifs conserved in a class of microorganisms, can be sensed by toll-like receptors (TLRs) and other pattern recognition receptors (PRRs) to activate innate immune responses, which effectively protect the host from infection.^31^ LPS, as the prototypical PAMP, can be recognized by multiple host receptors, including TLRs, PRRs, and nucleotide-binding oligomerization domain-like (NOD-like) receptors.^32^ The LPS-PRRs/NOD-like molecules activate inflammasome to produce proinflammatory cytokines,^33,34^ activating TNF-α and IL-1β, two of the most eminent inflammatory cytokines.^35^ Furthermore, LPS amongst five major Gram-negative bacteria have the ability to induce the production of NF-κB and proinflammatory IL-8 in a TLR4-dependent manner, suggesting that the pathogenesis of bacterial enhancement of chronic inflammatory diseases may be related to its serotype-specific LPS response.^36^

Apparently, LPS exhibits a crucial role in regulating the interaction of bacteria with their host, is the main cause of tissue degeneration and chronic damage. LPS induces respiratory tract infections by regulating epithelial-mesenchymal transition (EMT)-mediated airway remodeling.^37^ The mutations of LPS can result in attenuated virulence.^38,39^ Caffeine alleviates the excessive inflammatory response caused by *P. aeruginosa* infection by inhibiting the activation of LPS-mediated TLR4/MyD88/NF-κB/miR-301b signaling pathway, and improves lung tissue injury.^40^ Notably, LPS mutations confer bacteria gain tolerance to phage infection.^41^ Taken together, in addition to the direct interaction with the host PRRs receptors, LPS may use its unique molecular features to adjust bacterial pathogenesis and damage host immune defense, ultimately benefiting the fitness and invasive strength.

#### OMVs are important part of virulence platforms

OMVs are bacterial components that can be released spontaneously to the environment during growth by many Gram-negative bacteria. Bacterial-derived OMVs have been characterized as a novel secretion mechanism that can deliver a variety of bacterial proteins and lipids into host cells without direct contact with host cells.^42–44^ OMVs can package and enrich a wide variety of proteins and nucleic acids, including lipoproteins, periplasmic proteins (*E. coli* cytolysin A, enterotoxigenic *E. coli* heat-labile enterotoxin, and *Actinobacillus actinomycetemcomitans* leukotoxin), plasmid containing chromosomal DNA fragments, phage DNA, virulence factors (LPS, alkaline phosphatase, phospholipase C, β-lactamase, and Cif et al.^45,46^ *P. aeruginosa* secretion of OMVs have been implicated in many virulence-associated behaviors, including the acquisition of drug resistance, the regulation of bacterial density and host immune escape.^47–51^ Mechanistically, *P. aeruginosa* secretes OMVs to deliver virulence factors and sRNAs into lung epithelial cells through the diffusion the mucus layer.^44,47,52–55^ Some studies also illustrate that OMVs could lead to an increased hydrophobicity of cell surface, resulting in enhanced ability to form biofilms.^56^ OMVs is controlled by quorum-sensing systems, which enable bacteria to colonize and immune escape.^54,57^ Interestingly, OMVs are naturally immunogenic and self-adjuvation, making them have potential to be developed as antibacterial vaccine, such as OMV vaccine for *Neisseria meningitidis.*^58–60^ Therefore, OMVs are not only an important functional constitute, but also a potential biotechnological engineering carrier for vaccination or drug delivery. More details about virulence factors and their associated treatment strategies in *P. aeruginosa* are listed in Table 1 (45, 82–100).

**Table 1:** The major pathogenesis factors of *P. aeruginosa* and therapeutics

Pathogenic factor	Features and biological role	Therapeutic intervention	Vaccine availability
Proteases	*P. aeruginosa* secreted proteases include elastase A, elastase B, large protease, protease IV, alkaline protease, Pseudomonas small protease, MucD, and *P. aeruginosa* aminopeptidase. They exhibit high proteolytic enzyme activity that damages host tissues by degrading proteins.	Protease inhibitors	Preclinical^369,392,408^
Toxins	*P. aeruginosa* produces a variety of extracellular toxins, including pigments, phytotoxic factors, hydrocyanic acid, phospholipase, protein convertase, enterotoxin, exotoxin, and mucus. These exotoxins can cause leukopenia, acidosis, liver necrosis, pulmonary edema, circulatory failure, renal tubular necrosis and bleeding, and many other serious damages.	Bacteriophages	Preclinical^410,415,436^
LPS	LPS is an integral component of cell envelope. It is the major virulence factor of *P. aeruginosa* and can be recognized by host pattern-recognition receptors to initiate inflammation and immunity response.	Antibody	Phase III^380,389,425^
Pili and fimbriae	Pili and fimbriae are the major adherence factors. They contribute to the adherence and motility of *P. aeruginosa* in host.	Phages^380,422,424^	None
Flagella	The main protein component of flagella is flagellin. Flagella provide motility and chemotaxis toward specific substrates and provide the ligand for clearance by phagocytic cells.	Bacteriophages	Phase III^366,393,410^
Leukocidin (cytotoxin)	They are secreted by the typical secretion system (e.g., ExoU secreted by Type III secretion system) and are the main cytotoxin targeting lymphocytes and neutrophils.	Natriuretic peptides^376,380^	None
Siderophores	There are two siderophores produced by *P. aeruginosa*: pyoverdine (formerly called fluorescein) and pyochelin. In addition to the iron needs, siderophores can support other virulence factors production by transferring iron, such as biofilms and toxic themselves.	Antibiotic-siderophore^387^	None
Urease	Urease enzyme is a virulence factor (limited extent) of *P. aeruginosa*. It can hydrolyze urea to produce ammonia and carbon dioxide (CO_2_). It is associated with urinary tract infection.^378,427^	None	None
Outer membrane proteins	The outer membrane contains a large number of outer membrane proteins. These protein members are involved in the transportation of amino acids and peptides, the absorption of antibiotics, and the transportation of carbon sources. They are essential for bacterial adherence, virulence secretion, and host recognition.	Potential receptor for the internalization of host	Phase I^372,456,457^

## Secretion systems, an integral part of virulence platforms and mechanisms

**Fig. 2:**
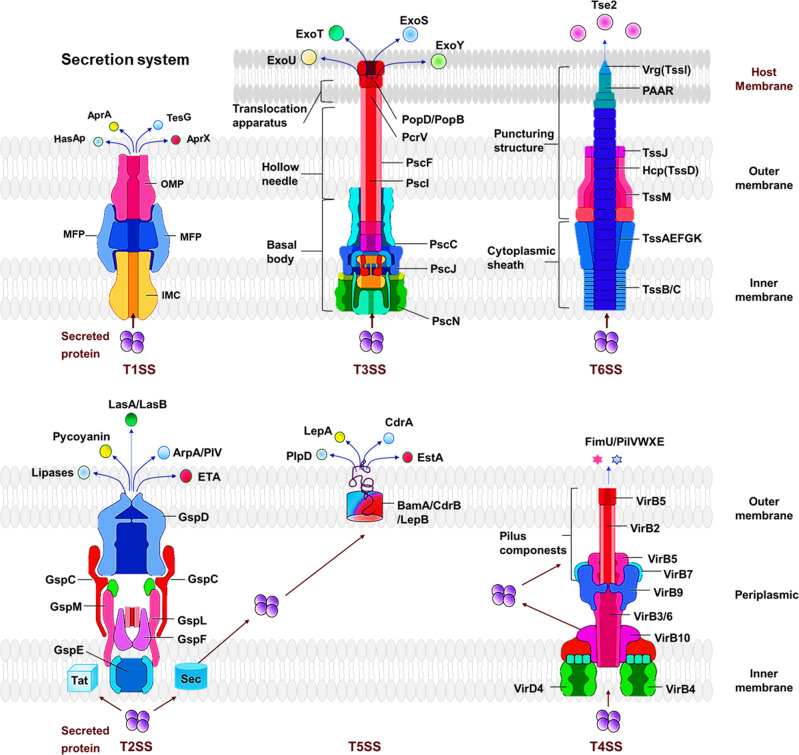
Protein secretion systems in *P. aeruginosa*. The secretion systems are divided into two major classes, one-step secretion system (T1SS, T3SS, T6SS) and two-step secretion system (T2SS, T5SS). One-step secretion system exoproteins are directly absorbed into the cytoplasm through their cognate secretion mechanism. In contrast, the exoproteins of two-step secretion system are first exported to the periplasm through the Sec or Tat system, and then crossing outer membranes through specific secretion mechanisms

Gram-negative pathogens cause various types of nosocomial infections, and secreted virulence factors often mediate their interactions with the host.^61^ Bacteria can modulate the host immune response through the secretion system for secreting virulence factors into host cells, which facilitates immune escape and enable bacterial colonization.^62^ Currently, 6 types of secretion systems (T1SS to T6SS) have been identified in *P. aeruginosa*. Based on the secretion routes of transport proteins, the secretion systems are divided into two major classes, one-step secretion system (T1SS, T3SS, T4SS, and T6SS) and two-step secretion system (T2SS and T5SS). The one-step secretion system directly secretes proteins from bacterial cytosol to the surface, while the two-step secretion system requires a brief periplasmic stay of the secreted proteins on the export way and then releases the proteins to outside environments of the bacterium (Fig. 2).

### One-step secretion systems

#### T1SS

Two different T1SS types in *P. aeruginosa* elucidated, Apr secretion system and Has secretion system.^63^ The Apr secretion system consists of three major components: AprD (ATP-binding cassette transporter, ABC transporter), AprE (adaptor), AprF (outer membrane factor, OMF), and secretes two proteins: AprA (alkaline protease), and AprX (protein with unknown function).^64^ T1SS is found in a variety of bacteria (*P. aeruginosa* *Salmonella enterica*, *Neisseria meningitidis*, and *E. coli*).^65^ T1SS major transport proteins (such as proteases and lipases). The substrate protein containing a C-terminal uncleaved secretion signal were recognized by the ABC transporter, were directly transferred across bacterial inner and outer membranes in a one-step process.^66^ The Has secretion system is composed of HasD (ABC transporter), HasE (adaptor), HasF, and OMF.^67^ Has secretion system participates in iron regulation by secreting an extracellular haem-binding protein (hasAp).^68^ Thus far, data relating to T1SS is very limited and its function in pathogenesis and significance for bacterium physiology and fitness are largely unknown, requiring further elucidation in order to know whether it has potential important functions.

#### T3SS

The T3SS of *P. aeruginosa*, playing a key role in virulence like quorum sensing, was first discovered in 1996^69^ and is one of the most extensively-studied secreted toxins with increasing evidence for its important virulent effects.^70^ The T3SS regulon comprises five distinct operons, including the *pscN* to *pscU*, the *exsD*-*pscB* to *pscL* operons, the *popN*-*pcr1*-*pcr2*-*pcr3*-*pcr4*-*pcrD*-*pcrR* operon, the *pcrGVH*-*popBD* operon and finally the *exsCEBA* operon.^71,72^ The five distinct operons play important roles in the biogenesis and translocation mechanisms of type III secretions. Structurally, the T3SS, similar to a molecular syringe, comprises five components: the needle complex, the translocation apparatus, the regulatory proteins, the effector proteins, and the chaperones.^73^ T3SS secretes virulence effectors (ExoS, ExoT, ExoY, and ExoU) into the eukaryotic host cells to disrupt intracellular signaling and ultimately causing cell death.^74–76^ Many bacterial factors regulate T3SS genes of *P. aeruginosa*. The MgtC and OprF of PAO1 regulate T3SS and ExoS-induced host macrophage damage.^77^ The T3SS is positively regulated by PsrA,^78^ HigB,^79^ Vfr,^80^ and DeaD,^81^ but negatively regulated by MexT,^82^ AlgZR, GacAS/LadS/RetS,^83–88^ and MgtE.^89^ No only for *P. aeruginosa*, the T3SS is a highly important secretion mechanism for Gram-negative bacterial invasion factors, which may also facilitate the bacterial evasion from the host immune responses to establish invasion, colonization, replication, and spread.

#### T6SS is a novel, important virulence machinery

In *P. aeruginosa*, T6SS is a newly identified powerful system with diverse and vital functions in virulence, bacterial interaction, and competition with the environmental microorganisms.^90^ Initially, the genome of *P. aeruginosa* was thought to constitute three gene clusters called Hcp Secretion Island (HSI) encoding T6SS components, which are later renamed H1-T6SS to H3-T6SS,^91,92^ with ~15–20 genes for each of them.^93^ In addition, the apparatus of T6SS, consisting of 13 core components, is divided into a baseplate-like structure, a sheathed inner tube assembled from the baseplate-like structure and a trans-membrane complex.^94^ The assembled T6SS appears to be an inverted phage tail, with the Hcp (hemolysin coregulated protein)/Vgr (valine-glycine repeat protein) complex forming the distal end of the cell-puncturing device.^95^ The sheath transits the effectors into targeted cells by a contraction-based mechanism.^96^ Furthermore, ClpV, an AAA+ family ATPase of T6SS, also provides the energy necessary to drive the secretory apparatus.^97^

Mechanically, the secretion process by T6SS needs other elements; for example, the H2-T6SS machinery to deliver the novel antibacterial toxin Tle3 requiring a cytoplasmic adaptor Tla3.^98^ The GacAS/Rsm regulates T6SS (H1-T6SS and H3-T6SS) by activating RsmY/Z to inhibit the binding activity of RsmA/RsmN to fha1/tssA1.^99^ H3-T6SS secrete TseF to facilitate the import of the PQS-Fe^3+^ complex into cells by incorporating it into OMVs with *Pseudomonas* quinolone signal (PQS).^100^ Interestingly, it was found that the quorum sensing (QS) system plays an important role in the expression of this secretion system. In *P. aeruginosa*, QS differentially regulates three loci of encoding T6SS (HSI-I, HSI-II, and HSI-III) by LasR and MvfR.^101^ The QS systems regulator of Las and Rhl controlls the expression of H2-T6SS in PAO1 strains^102^ and the QS regulator of MvfR directly modulates the expression of multiple proteins, including virulence factors and other regulators in PA14,^103^ respectively.

The H1-T6SS-dependent substrates have a broad research foundation, while only little is known about functional roles of H2-T6SSs and H3-T6SSs due to the limited substrates available for research.^104^ With collaboration efforts, we characterize H2-T6SS-dependent secretomes that are related to copper (Cu^2+^)-binding effector azurin (Azu).^104^ Azu and H2-T6SS are both inhibited by CueR while induced by low levels of Cu^2+^. Furthermore, our studies reveal an Azu-interacting partner OprC, which is a Cu^2+^-specific TonB-dependent outer membrane transporter and is also modulated by CueR. The Azu-OprC-mediated Cu^2+^ transport network may contribute to *P. aeruginosa* virulence. Our follow-up studies indeed illuminate that *oprC* dampens host response in cells and mice to *Pseudomonas* infection by potently enhancing Quorum-Sensing-associated virulence.^105^

In addition, we recently describe a function for H2-T6SS of *P. aeruginosa* for specific delivery of AmpDh3 (a paralogous zinc protease) to the periplasm of a prey bacterium upon contact.^106^ AmpDh3 can hydrolyze peptidoglycan located on the cell wall of the prey bacterium to induce prey cell death, which serves as evolutionary advantage for *P. aeruginosa* in a competitive environment.

In spite of the relative short time since discovery of T6SS, the progress in understanding the potent virulence pathway is fascinating and fast-moving, which may define many unknown functions that can be attributed to T6SS’ virulence and indicate new ways for treating patients who suffer the most severe disease and difficult to treat.^107^

#### T3SS and T6SS in cooperation for regulating host and bacterial responses

**Fig. 3:**
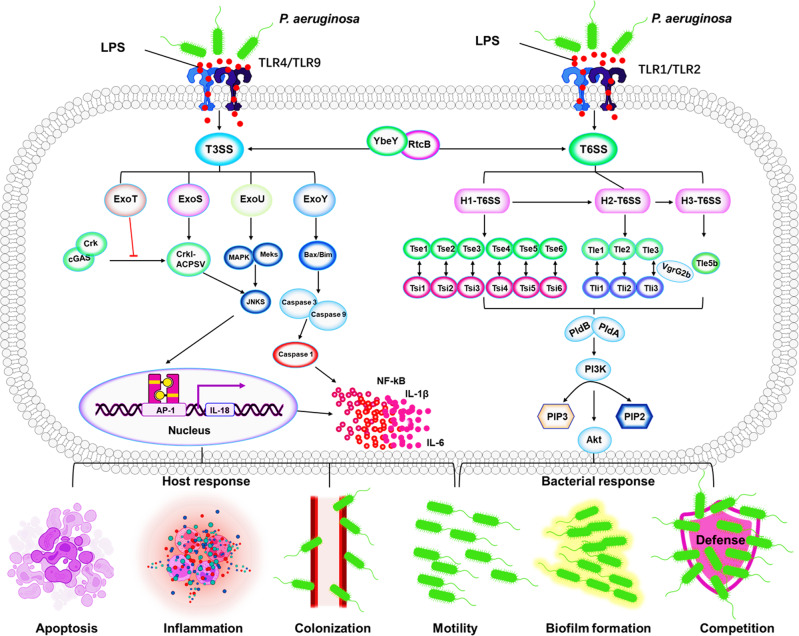
Mechanisms of T3SS and T6SS in regulating bacterial pathogenesis and host responses in *P. aeruginosa*. LPS is recognized by TLRs (TLR1/2 or TLR4/9) and then activates T3SS and T6SS*.*^479^ T3SS and and T6SS represent a critical network in regulating bacterial behaviors (growth, biofilm formation, and competition) and host defense (host cell apoptosis, inflammatory response, colonization, and motility). T6SS and T3SS interaction and inter-conversion are regulated by RtcB and YbeY. ExoS/ExoU induce *P. aeruginosa-afflicted* host cell apoptosis and colonization by targeting JNKS signal pathway. ExoY/ExoT reduces inflammasome activity through inhibition of bacterial motility to dampen NF-κB and caspase-1 activation. T6SS is a powerful antibacterial weapon that can be injected through multiple effectors to compete with other bacteria and allows *P. aeruginosa* colonization and biofilm formation^108,109^

T3SS and T6SS are indicated to regulate host and bacterial responses, including host cell apoptosis, inflammatory response, colonization, motility, biofilm formation, and bacterial competition/interaction^108,109^ (Fig. 3). Interaction regulation and inter-conversion of T6SS and T3SS may be especially helpful for coping with complex environmental pressures.^110^ The switch between T6SS and T3SS is directly regulated by the RNA-binding protein RtcB controlling colonization, establishment, and pathogenicity in *P. aeruginosa.*^111^ YbeY regulates T3SS and T6SS secretion systems and biofilm formation by controlling RetS.^112^ The function of T3SS is regulated by various regulators, including four main regulators genes (exsA, exsC, exsD, and exsE), which is involved in the transcription activation of the aforementioned classical effectors (exoS, exoT, exoU, and exoY).^72,113^ ExoS is a 48.3 kDa protein containing 453 amino acid length. It has been reported early that ExoS participates in host cell apoptosis via its GAP region or ADP-ribosyltransferase (ADPr) activity.^114,115^ Furthermore, the ExoS possesses ADPRT activity, which induces *P. aeruginosa*-afflicted host cell apoptosis by targeting a variety of Ras proteins.^116^ ExoU is the longest *P. aeruginosa* effector containing 687 amino acids (73.9 kDa).^117^ ExoU is the most acutely cytotoxic among the four effector proteins because it can induce rapid cell death and is considered to be the main driver of the cytotoxic phenotype.^118^ ExoU dysregulates the host’s innate inflammatory response by poisoning and killing immune cells, including macrophages, neutrophils, epithelial cells, and endothelial cells, allowing bacteria to persist, proliferate, and spread, and ultimately leading to sepsis, Alzheimer’s disease, acute respiratory distress syndrome, *etc*.^114^ Mechanistically, ExoU transiently represses Capase 1 and NLRC4 inflammasome activation, inhibiting the release of IL-1β, IL-18, and proinflammatory DAMPs, and thereby suppressing the host immune response.^119,120^ In addition, ExoU can activate AP-1 transcription factors to increase IL-8 production and induce tissue-damaging inflammatory by JNK/MAPK) pathway.^121^ ExoT containing 457 amino acids (48.5 kDa) has GAP and ADPRT activities, and can induce host cell apoptosis by targeting Crk proteins phosphorylation of p38β and JNK induces apoptosis, which subsequently interferes with integrin-mediated survival signaling via destroying the stability of focal adhesion sites.^122^ Notably, recent studies have shown that *P. aeruginosa* ExoT induces G1 cell cycle arrest in melanoma cells, suggesting its potential for regulating the cell cycle.^123^ ExoY is a 378 amino acid protein with a molecular size of 41.7 kDa, detected in 80–100% of *P. aeruginosa.*^124^ ExoY plays a direct role in immune escape by inhibiting TAK1 activation, which is a key factor in the TGF-inducible pathway that directly modulates immune responses, contributing to *P. aeruginosa* survival and infection severity.^125^ In addition, ExoY regulates host inflammatory responses by delaying activation of NF-κB and caspase-1.^126^

The T6SS components and their effectors are diverse and complex beyond bacterial-cell targeting. T6SS systems have been detected in ~200 Gram-negative bacteria, including *P. aeruginosa.*^109^ To compete for survival in the living environment, H1-T6SS kills other bacteria by injecting Tse2 effector molecules into other target bacteria possessing antibacterial activity and providing advantages for *P. aeruginosa* growth. In addition, to protect itself from Tse2 toxins, *P. aeruginosa* also produces the antitoxin Tsi2.^127^ Similarly, H1-Tse1 and Tse3 are injected into the periplasm of other bacteria to hydrolyze peptidoglycan, which can be counteracted by periplasmic immune proteins Tse1 and Tse3.^128^ Tse4, Tse5, Tse6, and Tse7 that also show antibacterial activity are associated with homologous immunity.^129^ The phospholipase D enzymes PldA and PldB of Tle5 were injected to other bacteria by H2-T6SS and H3-T6SS to exert antibacterial activity.^130^ Further, VgrG2b is injected into epithelial cells by H2-T6SS, in which it targets the γ-tubulin ring complex component (γ-TuRC) and promotes the recruitment of PI3K at the apical membrane.^131,132^ Moreover, PldA/B targets the host PI3K (phosphoinositide 3-kinase)/Akt pathway to remodel the PIP3 (phosphatidylinositol-3,4,5-triphosphate) and actin in the apical membrane, which is essential for successful colonization of the host by *P. aeruginosa.*^133^ T6SS-related genes hcp1/hcp3 and lasR were significantly higher in the strong biofilm formation (SBF) compared to nonbiofilm formation (NBF) groups, which contributes to the biofilm production by *P. aeruginosa.*^90^ Collectively, T6SS is a powerful antibacterial weapon that can be injected with many different effectors to compete with other bacteria and allow *P. aeruginosa* colonization and biofilm formation.

### Two-step secretion systems

Different from the One-Step secretion, Two-step secretion requires a brief periplasmic phase of the secreted proteins on the export route before being exported to the outside of the cell through general export pathways, which plays an important role in the transport of periplasmic and outer membrane proteins.

#### T2SS

The function of T2SS is one of the less characterized secretion systems and is thought to secrete folded proteins from the periplasm.^134^ Two different pathways exist in T2SS: the general secretory (Sec) and twin-arginine translocation (Tat).^135^ The secreted proteins are first transited through the inner membrane, stays briefly in the periplasm and then secreted into the extracellular environment.^136,137^ The Sec pathway consists of a protein targeting component, a motor protein, and a membrane integrated conducting channel called SecYEG translocase, the secreted proteins with a SecB-specific signal sequence might be guided to the periplasm or the extracellular environment.^138^ The twin-arginine translocation (Tat) pathway of Gram-negative consists of TatA and TatB, which can decide whether the secreted is retained in the periplasm or translocated to the extracellular space with a twin-arginine motif.^139^ Functionally, T2SS participates in the secretion of guanylate cyclase ExoA, proteases lasA/B and multiple other factors and many of which have emerged as potential therapeutic targets.^140,141^

#### T5SS

The T5SS of *P. aeruginosa* is composed of five subtypes (type Va to Ve) and exports the proteins across the inner membrane via the Sec pathway.^142^ The proteins of the V-type secretion system are often referred to as autotransporters (ATs). Typically, the T5SS consists of only one polypeptide chain with a β-barrel translocator domain in the membrane, and an extracellular passenger or effector region.^143^ Under the regulation of the Bam complex (β-barrel assembly mechanism) and TAM complex (translocation and assembly module), outer membrane proteins fold to form a β-barrel conformation and insert into the outer membrane.^144^ T5SS secretes a variety of proteins, including EstA, LepB, and LepA. EstA has esterase activity and is involved in rhamnolipid production and biofilm formation.^143^

The type Vb secretion system comprises two distinct polypeptide chains encoded in one operon, therefore, it is also known as the dual-partner secretion system (TPSS) containing the passenger domain (TpsA) and the β domain (TpsB).^145^ TpsA has a TPS secretion motif and a functional/catalytic domain and the TpsB is a 16-chain OM-integrating β-barrel protein with two periplasmic POTRA (polypeptide transport-associated) domains.^146^ The Vc-type secretion system forms a highly intertwined trimeric structure and is therefore also known as a trimeric autotransporter adhesin (TAA).^147^ The C-terminal β-barrel domain of the Type Vd consists of 16 β-strands, similar to the β-barrel of TpsB proteins.^145^ The Ve-type ATs share obvious similarities with Va-type ATs, the main difference is that Ve-type ATs have an inverted domain order, with the β-barrel at the N-terminus and the passenger at the C-terminus.^148^ Despite these preliminary studies, there is much more to be learnt regarding T5SS for the physiology and virulence in *P. aeruginosa*.

#### T4SS

T4SS is a multisubunit cell-envelope-spanning structure that can transfer protein and nucleoprotein complexes across membranes, which is related to horizontal gene transfer-mediated antibiotic resistance, adaptation, evolution, and virulence.^149^ T4SS in bacteria is divided into the IVA-type secretion system represented by *Agrobacterium tumefaciens* VirB/VirD4 and the IVB-type secretion existed as *Legionella pneumophila* Dot/Icm system. Those distinct from the above are classified as “Other T4SS”, which contain genomic islands pKLC102 and PAPI in *P. aeruginosa* and are less characterized than other two types.^149^ *P. aeruginosa* T4SS comprises abundant major pilin subunits, PilA, and low abundance minor pilins FimU and PilVWXE. Both major pilin and minor pilins are processed by the pre-prepilin peptidase, PilD to exert function. T4SS assembly system is evolutionarily associated with T2SS contributing to the process of assembly and disassembly of pili. Minor pilins can impact assembly, retraction, extension, and adhesion.^150^

Again, the chief function of T4SS is horizontal genetic transfer (HGT) between different microorganisms and potentially relating to pathogenesis.^151^ For the genetic island containing T4SS, there is a discrepancy: whether a conjugation mechanism exists but this is likely related to differences between strains.^152^ Nevertheless, there are relatively limited studies of this system compared to other secretion systems such as T3SS. We will further discuss the potential role of T4SS in host response context, initiating/activating inflammasome independent on nucleotide oligomerization domain (NOD)-like receptor (NLR) family CARD (C-terminal caspase recruitment domain) domain-containing protein 4 (NLRC4),^153^ in addition, the virulence factors and their associated secretion systems in *P. aeruginosa* were summarized in Table 2.

**Table 2:** The virulence factors and their associated secretion systems in *P. aeruginosa*

Secretion systems	Virulence factors	Features	Pathogenicity	Inhibition	Interaction\regulation
T1	Alkaline protease	Adherence and Colonization^342^	Corneal infections	APRin^363^	Ca^2+^^372^
	HasAp	Proliferation^375^	Scavenges heme	Metal complexes^382^	hasR
T2	LasB (elastase)	Biofilm formation^353^	Antibiotic resistance	Ag-phendione, Cu(phendione)^395^	IL-1β^438^, LasA^384,395,397,404,438,451,458^
	LasA	Biofilm formation and Colonization	Antibiotic resistance^459^ and Corneal infections^370^	Satureja khuzistanica essential oil^339,343,369^	LasD
	Phospholipase C (PlcH, PlcN)	Host inflammation regulation	Cell hemolysis and Neutrophil activity^344^^,354^	Global regulator Anr	Ca^2+^, Pi, Zn^+^, PlcR1, PlcR2, IL-8^375,385,420,421,440^
	PrpL protease	Biofilm formation	Wound healing and Corneal virulence^403^	–	Iron, Pvds, AprA, LasB^434,447^
	Lipases (LipA, LipC)	Motility, Biofilm formation and Rhamnolipid production	Enhanced PLC-induced 12-HETE and LTB4 generation	DsbC protein^381^	PmrA/PmrB^401,411^
	ToxA	Colonization and Proliferation	Impairment of host defense, Inhibition of protein synthesis and Interference with cellular immune functions	Norepinephrine^340^	Iron and PvdS^404^
	LapA (via Hxc T2SS)	Biofilm formation and Localization adhesin	Antibiotic resistance^346^	–	LapF, c-di-GMP^374,377,405,419^
	Mep72	–	–	–	PopD, PcrV, ExoS, FliD
T3	ExoS	Avoid phagocytosis and Kill the host cell	Inhibition of autophagy and mTOR, ROS Production and Induces apoptosis	Cinnamaldehyde and Carvacrol and honey^413,417^	RpoS, Rac^116,418,429,444,449^
	ExoY	Enhance acute pathogenicity and infection	Impairs endothelial cell proliferation and vascular repair^116,418,429,444,449^	Aspirin	GSH, NF-κB, AP-1, TAK1
	ExoT	Inhibition of internalization by epithelial cells and macrophages	Inhibits lung epithelial wound repair^386,398,399,407,437^	–	RhoA, Rac1, Cdc42
	ExoU	Overexpression efflux pumps and AmpC β-lactamase genes	Antibiotic resistance and Augments neutrophil transepithelial migration	Arylsulfonamides^388,409,426^	NF-κB, IL-8/KC
	ExsE	Negative regulator of type III secretion gene^430,455^	–	–	T3SS
T5	Esterase (EstA)	Enhanced rhamnolipid production, Cell motility and Biofilm formation	–	–	ExsC, ExsD^443,448^
	CupB5	Activates alginate overproduction	–	–	AlgU, MucA, TpsB4\LepB^381,390^
	Exoprotease (Lep)	Producing pyocyanin	–	–	Iron^396^
T6	Tse1 (amidase)	Defense against other organisms	Lysis of cells	Tsi1	Tsi1^360^
	Tse2	Defense against other organisms	Lysis of cells	Tsi2	Tsi2^431^
	Tse3 (muramidase)	Defense against other organisms	Lysis of cells	Tsi3	Ca^2+^, Tsi3^416,445^
Other secretion systems	HCN	–	–	–	Glycine, AlgR, LasR and RhlR^382,383^
	Pyocyanin	–	Interferes with multiple cellular functions	Baicalin, 4-aminopyridine	BrlR, PhzM^383^^,387,397^
	Pyoverdine	High-affinity iron uptake from transferrin and lactoferrin	Keratitis	Phenylthiourea	PvdP tyrosinase^439,446^
	Rhamnolipids	Biofilm formation	Potentiate aminoglycoside antibiotics	H_2_S	Oxygen, Polyhydroxyalkanoate^371,418,421,422^
	Alkyl quinolones	Bacterial communication and quorum sensing	–	–	PqsR, LysR^433^
	N-acyl homoserine	Biofilm formation	–	–	Propolis^379^
	Lactones	Biofilm formation	Accelerate host immunomodulation	Th1 and Th2 cytokines^401^	–

## Virulence regulatory systems

The regulation of all these virulence factors is cell density-dependent through release of autoinducers of critical quorum sensing (QS) (e.g., Las, Rhl, Pqs, and Iqs), a mass communication system.^154^ QS may help large population fitness by a hierarchical signal pattern to survive in fierce host environments and thrive, leading to persistent infection in individuals with cystic fibrosis, which cannot be completely cured even with tremendous progress in drug development, drastically improved medcare systems and living conditions.^155^ Hence, QS systems along with some other critical virulence factors, such as six types of secretion systems (of toxic molecules), two-component systems (TCSs), have become an intense interest in mechanistic understanding of this bacterium.

### Quorum sensing is the most-studied systems for regulating gene expression and virulence

#### Roles and acting mechanisms of QS

QS describes a method that is widely utilized by bacteria for cell–cell mass communication. Both Gram-negative and -positive bacteria detect the local population density by sensing chemical signals and coordinate gene expression and group-beneficial behaviors.^156,157^ Bacteria produce autoinducer or quoromone as diffusion signaling molecules and release into the environment for communication. Once the population reaching a threshold, the autoinducers activate their cognate receptors to directly or indirectly induce gene expression.^158^ Over the past two decades, QS has been extensively studied as a potential target for ‘antivirulence agents’, which may be harnessed to counteract bacterial virulence via a noncytotoxic mechanism as alternatives of traditional antibiotics.^159,160^

Another essential function for QS in *P. aeruginosa* is to regulate the production of multiple virulence factors, such as extracellular proteases,^161^ iron chelators,^162^ efflux pump expression,^163^ biofilm development,^164^ swarming motility,^165^ and the response to host immunity.^166^ As a model organism, *P. aeruginosa* serves as one of the most suitable bacteria to study the fundamental mechanisms of QS signaling regulating virulence and search for chemical agents to block the QS system.^167,168^

#### Interaction between quorum-sensing systems and environments

Bacteria communicate with each other through the QS, which acts as a global regulatory system by directly or indirectly controlling the expression of genes, has been the central point of research.^157^ For example, scientists have made significant efforts in understanding the interactions between all the four QS systems and also how environmental cues may affect gene expression and function of the QS. Two canonical *N*-acyl L-homoserine lactone (AHL) based (Las and Rhl) and two 2-alkyl-4 quinolones (AQ) based (Pqs and its precursor Hhq) signaling systems.^70^ These systems connect and coregulate each other. Rhl and Pqs were positively regulated by the Las system, while Rhl represses Pqs and Pqs augments Rhl.^169^ For example, in response to various nutritional and environmental stimuli, the regulatory relationship between Rhl and Pqs systems can change independently of Las.^170^

The activation of QS genes generally requires a large number of regulatory factors to control receptor expression or function, and/or to coregulate some QS-controlled target genes since the QS systems are functional diverse, organizational complex, and consisting of a spectrum of key regulators (including *rpoS, vqsR, mvfR, rhlR, rsaL* et al.).^171^ RpoS indirectly plays a subtle role in activating *lasR* and *rhlR* expression and modulating ~40% QS-controlled gene expression during the stationary phase.^172^ VqsR controls the production of AHL signaling molecules and virulence factors by inhibiting the LuxR-type regulator QscR, which represses *lasI* expression to regulate the timing of QS activation.^173^ VqsM positively regulates the QS systems by controlling several relevant QS regulators ranging from QS to antibiotic resistance, and *P. aeruginosa* pathogenicity.^174^ MvaT and its homolog MvaU control the magnitude and timing of QS-dependent gene expression,^175^ which have a massive impact on all three QS systems by directly regulating *lasR, lasI, rsaL, pprB, mvfR*, *algT/U, mexR*, and *rpoS.*^176^ RsaL binds to the *lasI* promoter and prevents LasR-mediated activation, regulates *las* signaling,^177,178^ and modulates the activity of PqsH and a recently identified regulator, CdpR, which are required for PQS synthesis.^179^ AmpR activates QS-regulated genes to positively influence acute virulence, while negatively regulating biofilm formation.^180^ CdpR negatively modulates bacterial virulence by impacting the expression of *pqsH*, which is positively regulated by LasI and VqsM along with QscR and RsaL.^181^ We also recently found that BfmR/S and/or its variants modulate the *rhl* QS system in *P. aeruginosa.*^182^ Crc regulates *rhl* QS by promoting Hfq-mediated suppression of lon gene expression.^183^ More recently, our laboratory delineated that AnvM is a critical regulator of virulence in *P. aeruginosa* by directly interacting with the QS regulator MvfR and anaerobic regulator Anr.^184^ The aforementioned discoveries have highlighted the rapid progress in understanding diverse, heterogenous regulatory mechanisms of QS coordinated by seemingly a large number of unprecedented factors, which may finally characterize powerful, versatile regulatory proteins or systems to be applied to better control the notorious pathogen.

Several factors (such as QscR and QteE) have been identified to regulate the activation threshold of quorum-regulated genes, which control QS activation timing through additional homeostatic mechanisms.^174^ In addition to the QscR mentioned above, QteE also blocks QS-regulated genes’ expression by preventing LasR and RhlR accumulation and blocking Rhl-mediated signaling.^185^ Moreover, QslA is found to interact with LasR.^186^

Based on extensive studies to date, we may presume that QS may be one of the most important regulatory systems in *Pseudomonas* contributing substantially to bacterial physiology, adaptation, and pathogenesis. Although, various studies have tested the ideas of targeting QS for potential therapy of bacterial infection, the effect of using QS-associated approach for treatment is unsatisfactory.^187^ In particular, there were only limited reports of in vivo treatments targeting QS.^188^ Hence, it is necessary to further study the fundamental role of QS in bacterial pathogenesis and identify new anti-virulence targets and approaches that would help develop urgently needed medicines for treating refractory infection in clinics for QS bearing bacteria.

### The two-component regulatory systems are critical gene regulators and virulence factors

The two-component systems (TCSs) are ubiquitous, complex signaling regulators that play vital roles in bacterial survival, metabolism, and virulence.^189^ As a versatile opportunistic pathogen, *P. aeruginosa* virulence network is tightly controlled by a growing number of TCSs.^190^ In general, a TCS pair of genes consists of a membrane-bound sensory histidine kinase (HK) and a cytoplasmic response regulator (RR). In *P. aeruginosa*, 64 HKs and 72 RRs have been identified.^191^ More than 50% of the TCSs and their corresponding HKs are linked to virulence and/or antibiotic resistance of *P. aeruginosa.*^192^ For instance, CzcR/CzcS is implicated in carbapenem-resistance;^193^ KinB/AlgB is involved in the alginate synthesis and virulence;^194^ and GacS/GacA is essential for pathogenicity.^195^ Remarkably, an attenuation in virulence behavior can be achieved by blocking TCS signaling. Goswami et al. reported that inhibition of HKs, especially Rilu-4 and 12, significantly reduced the production of virulence factors and toxins, and severely impacted the motility behavior of PA14.^195^

Our recent studies discovered a new copper-responsive TCS called DsbRS in *P. aeruginosa*, in which DsbS (sensor of histidine kinase) and DsbR (cognate response regulator) modulate gene transcription for disulfide bond formation (Dsb). DsbS (phosphatase) targets DsbR to interfere with the transcription of Dsb genes and help the bacterium cope with copper stress.^196^ Intriguingly, transcription factors can also regulate the behaviors of bacteria to adapt host environments; for instance, imidazole-4-acetic acid (ImAA) and its receptor HinK are recently implicated in the response of *P. aeruginosa* to histamine.^197^ These findings help understand the communication between *P. aeruginosa* and hosts to adjust bacterial health. We have summarized TCSs and their roles in controlling the key virulence factors in *P. aeruginosa* (Table 3).

**Table 3:** The composition and function of the two-component regulatory systems

Composition	Virulence factor	Function
PilSR	*Type IV pili*^460^	Twitching and Swimming Motilities
RocS1-RocR-RocA1	*CupC fimbriae*^461^	Biofilm formation/Antibiotic tolerance
FimS-AlgR, KinB-AlgB	*Alginate*^462^	Exopolysaccharide alginate synthesis
BfiSR	*Rnase G*^463^	Regulation sRNA levels
GacSA, RetS	*Exopolysaccharides (Pel, Psl)*^464^	Biofilm formation
RcsCB, PvrSR	*CupD fimbriae*^204^	Fimbriae assembly/Biofilm formation
BfmSR	*Edna*^465^	Bacteriophage-mediated lysis and DNA release/Biofilm formation
PprAB	Cup E fimbriae^466^	Fimbriae assembly/Biofilm formation
BqsSR	*Rhamnolipid*^467^	Quorum sensing-dependent biofilm
FleSR	*Flagella*^468^	Flagellar biogenesis
FimS-AlgR	*Type IV pili*^469^	Twitching motility/Biofilm maturation, Surface adhesion/Virulence
PA2573-PA2572	*ExoS*^469^	Virulence and antibiotic tolerance
TtsSR	*CbpE*^470^	Specific secretion of the CbpE chitin-binding protein
GtrS-GltR	*ToxA*^471^	The expression of exotoxin A and glucose catabolic enzymes
GacS-LadS-RetS, CsrA/RsmA	*Type III secretion*^472,473^	Virulence/Secretion systems/Biofilm formation, Quorum sensing/Motility
RsmA	*Elastase*^474^	Pyocyanin, rhamnolipids and elastase production
LadS	*ExoU*^475^	Virulence and the switch between acute and chronic infections
RocS1-RocR-RocA1	*ExoY, ExoT*^476^	Biofilm formation
CbrAB	*ExoT, ExoS*^477^	Metabolism, virulence, and antibiotic resistance
FimS-AlgR	*Regulated by PilY1*^469^	Virulence

The number of TCSs related to the virulence of *P. aeruginosa* has recently grown considerably, which may be highlighted by the multi-kinase networks containing multiple sensor kinases through crosstalk (network) to impact virulence.^192^ The networks include: (1) GacS network governs the switch between acute and chronic infections;^198–201^ (2) Roc network and Rcs/Pvr network control cup fimbriae production;^202–204^ (3) A complex network of five TCSs (PmrB, PhoQ, ColS, ParS, and CprS) regulates the aminoarabinose modification of lipopolysaccharide;^205–207^ (4) Wsp chemosensory pathway modulates biofilm formation and chronic infection;^208,209^ and (5) Chp/FimS/AlgR network involves biofilm formation and virulence.^210,211^ Although TCSs may control many functions, some of them may have unknown roles that require further studying. TCSs play a significant role in controlling either *P. aeruginosa* virulence or virulence-related behaviors (such as biofilm formation and antibiotic resistance). The functions of TCSs may contribute to the clinical significance exemplified by a more recently identified BfmRS TCS.^182^ Indeed, the plasticity of TCSs mediated by spontaneous mutations of BfmRS in patients has been assessed, and the data suggest that mutation-induced activation of BfmRS may be related to host adaptation by *P. aeruginosa* in chronic infections.^212^ Despite increasing identification of novel TCSs, several critical questions remain to be answered in future investigation: how multi-kinase networks process and integrate signals, which of the kinases plays a dominant role in multi-kinase networks, and how these TCSs interact with other systems, quorum sensing and secondary messenger signals to confer pathogenicity (e.g., cAMP and c-di-GMP).

### Small non-coding regulatory RNAs play roles in regulating gene expression and virulence

Small non-coding regulatory RNAs (sRNAs) range from 50 to 400 nucleotides in length.^213^ Since the discovery as part of a large group of transcriptional regulators in *Escherichia coli* in the 1960s, sRNAs are gradually recognized as key modulators to mount rapid responses when necessary and are encoded widespread in the bacterial genomes^213–215^ despite to different extent (the number of sRNAs in PAO1 is approximately twice higher than that in PA14). sRNAs play an essential role in bacterial pathogenicity and virulence mechanisms, such that participation in quorum-sensing regulation, ion metabolism, biofilm formation, stress responses, host cell invasion, and adaption to growth conditions.^216–218^ Hfq-dependent sRNAs are instrumental for modulating *P. aeruginosa* virulence.^219^ *rsmY* and *rsmZ* act as early responders to finely modulate bacterial cooperation under environmental stimuli to optimize population density.^220^

In *P. aeruginosa*, sRNAs can regulate bacterial metabolism instantly and precisely to establish successful infection in the hosts.^218^ A total of 573 sRNAs were detected in PAO1 and 233 sRNAs in PA14, indicating their quantity variation in different strains.^201^ Although 126 sRNAs are found in common to both strains, sRNAs can evolve rapidly, and many sRNAs exhibit strain‐specific or environmental-dependent expression.^221^ Interestingly, we recently revealed that sRNA PhrS may help generate CRISPR RNA (crRNA) for initiating bacterial immunity against bacteriophages, which is achieved through inhibiting Rho function on transcription-termination.^222^ Collectively, it would be intriguing to further understand the functions and underpinning mechanisms involving sRNA in this bacterium, which may identify novel pathway regulators and drug targets for controlling bacterial invasion.

## Acquisition of antibiotic resistance contributes to bacterial survival and thriving

The acquisition of drug resistance by *P. aeruginosa* depends primarily on multiple intrinsic and acquired antibiotic resistance mechanisms, including the biofilm-mediated formation of resistant and multi-drug-resistant persistent cells.^223^ Therefore, *P. aeruginosa* can quickly develop resistance to various antibiotics, including aminoglycosides, quinolones, and β-lactams.^224^

**Fig. 4:**
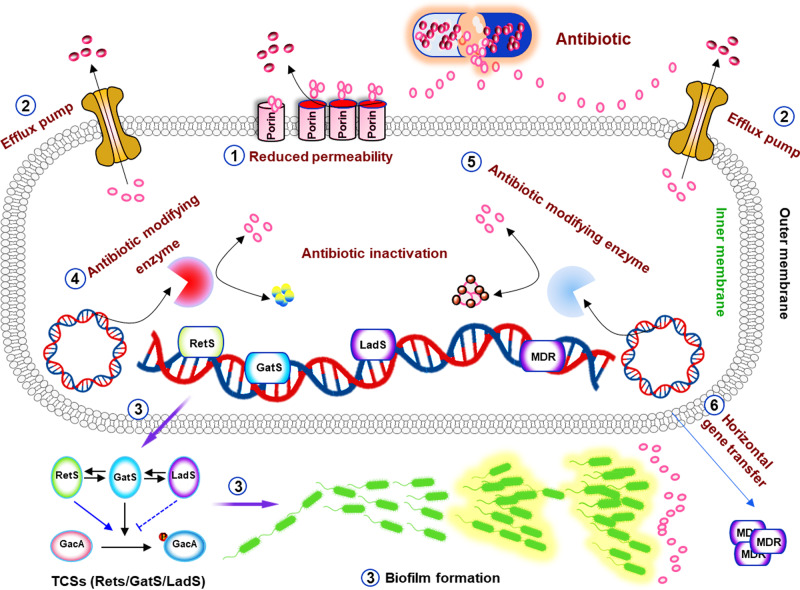
Mechanisms of antimicrobial resistance in *P. aeruginosa*. Mechanisms of antimicrobial resistance in *P. aeruginosa* can be divided into intrinsic antibiotic resistance (① outer membrane permeability, ② efflux systems, and ④antibiotic-modifying enzymes or ⑤ antibiotic-inactivating enzymes), acquired antibiotic resistance (⑥ resistance by mutations and acquisition of resistance genes), and adaptive antibiotic resistance (③ biofilm-mediated resistance). Alteration of outer membrane protein porins decreases the penetration of drugs into cells by reducing membrane permeability. The efflux system directly pumps out drugs. Drug-hydrolyzing and modification enzymes render them inactive. Similarly, some enzymes cause target alterations so that the drug cannot bind its target, resulting in drug inactivity. Antibiotic resistance genes carried on plasmids can be acquired via horizontal gene transfer from the same or different bacterial species,^225^ quorum-sensing signaling molecules activate the formation of biofilms, which act as physical barriers and prevent antibiotics penetrating the cell

In the course of long-term evolution, bacteria have developed a variety of ancient genetic resistance mechanisms that have significant genetic plasticity against harmful antibiotic molecules, enabling them to respond to various environmental threats, including possible harm (e.g., antibiotics, chemical compounds, and antimicrobial peptides) to their survival. The acquisition of antibiotic resistance with *P. aeruginosa* is quite diverse, and some primary mechanisms including outer membrane permeability, efflux systems, and antibiotic-inactivating enzymes will be addressed below^225^ (Fig. 4).

### Outer membrane permeability

To treat *P. aeruginosa* infections, most antibiotics need to penetrate the cell membrane to reach the intracellular compartment to function.^226^ The outer membrane of *P. aeruginosa* can act as a specific hurdle inhibiting antibiotic penetration. The outer membrane of *P. aeruginosa* is chiefly composed of bilayer phospholipid molecules, LPS and porins embedded in phospholipids. The outer membrane is responsible for the specific and non-specific uptake of extracellular substances relying on different porin functions, including non-specific porins (OprF), specific porins (OprB, OprD, OprE, OprO, and OprP), gated porins (OprC and OprH), and efflux porins (OprM, OprN, and OprJ).^227–230^ *P. aeruginosa* manipulates different porins to limit antibiotic penetration and increase antibiotic resistance. OprF promotes the formation and attachment function of *P. aeruginosa* biofilm to protect the bacterium against antibiotics.^231–233^ Mutations of specific porins OprD involving conformational features cause carbapenem resistance, a serious challenge for medical treatment practices.^234^ Outer membrane protein H (OprH) of *P. aeruginosa* enhances the stability of the outer membrane through direct interaction with LPS to regulate antibiotic resistance.^235,236^ Efflux porins (OprM, OprN, and OprJ) contribute to the active efflux of several antibiotics, including tetracycline, norfloxacin, and β-lactam antibiotics.^237^ These findings demonstrate that the elegant effects and diverse mechanisms by which porins determine antibiotic resistance.

In a separate account, in recent years, OMVs secreted by *P. aeruginosa* are shown to be able to transport multiple virulence factors, like hemolytic phospholipase C, mRNA, DNA, β-lactamase, alkaline phosphatase, to the host cytoplasm, which may be a part of pathogenic mechanisms of antibiotic resistance. OMVs may be fused with the host plasma membrane through receptor-mediated endocytosis. While OMVs are detrimental to the host by delivering antibiotic resistance molecules or enzymes (β-lactamase), they have been harnessed as alternative delivery vehicles for transporting treatments or vaccines for various diseases including infection and cancer.^47,238^

### Efflux systems for pumping off drug resistance factors

The toxic compounds derived from metabolism or antimicrobials inside bacterial cells are harmful to bacterial survival, and require mechanisms to expel. The efflux pump is a main, conserved mechanism to remove antibiotics. The efflux pump may upregulate virulence genes (QS) for enhancing antibiotic resistance and maintaining bacterial homeostasis. Currently, five components are described in the efflux pump family: ATP-binding cassette (ABC) superfamily, major facilitator superfamily (MFS), and multidrug, toxic compound extrusion (MATE) family, resistance-nodulation-division (RND) family, and small multidrug resistance (SMR) family.^239^ Among all efflux pumps, the RND efflux pump family has the strongest correlation with antibiotic resistance. Twelve RND family efflux pumps are identified in *P. aeruginosa*: multidrug efflux AB-outer membrane porin M (MexAB-OprM), multidrug efflux CD-outer membrane porin J (MexCD-OprJ), multidrug efflux EF-outer membrane porin N (MexEF-OprN) and multidrug efflux XY-outer membrane porin M (Mex XY-OprM) can increase resistance to a variety of antibiotics through efflux.^240^ MexAB-OprM is crucial for developing carbapenem-resistant *P. aeruginosa*, which is a lingering clinical issue.^237^ Upregulation of MexCD-OprJ is closely associated with increased resistance of most clinical strains to ciprofloxacin, cefepime, and chloramphenicol.^241^ Quinolones MexEF-OprN are overproduced by the QS deficiency due to kynurenine extrusion.^242^ Furthermore, the resistance of *P. aeruginosa* to aminoglycosides, fluoroquinolones, and zwitterionic cephalosporins depends on the efflux function of MexXY-OprM.^243^ These findings suggest that despite some similarity in substrates, their affinity of efflux pumps may vary greatly, displaying different extent of anti-antimicrobial activities.

Multiple lines of evidence suggest that the chief mechanism for *P. aeruginosa* success in infection is highly related to its stubborn resistance to antibiotics or other therapeutics, which is regulated by the efflux pump. Hence, targeting this mechanism such as inhibiting the critical efflux pump—mexAB-oprM or enhancing the repressor—mexR, will likely reveal new strategies to overcome antibiotic resistance mechanisms in the bacterium and achieve the improved treatment efficacy.^244^

### Antibiotic-inactivating enzymes that cleave enzymatic drugs

Antibiotics often contain chemical bonds (e.g., amides and esters), and bacteria can produce antibiotic-inactivating enzymes (hydrolase) to degrade or alter antibiotics, leading to antibiotic resistance.^245^ *P. aeruginosa* is highly resistant to various antibiotics including penicillin, cephalosporin, and aztreonam by producing extended-spectrum β-lactamases (ESBLs).^246,247^ In addition, the bacterium is also resistant to the cefazolin-tazobactam combination therapy via ESBL GES-6.^248^ Again, the ESBL of *P. aeruginosa* is thought to be the most significant mechanism in terms of countering antibiotics, which would be a major target for designing and developing the most potent antimicrobial drugs.

From enzymatic angles, *P. aeruginosa* can modify the amino groups and glycosidic groups of aminoglycoside antibiotics to produce antibiotic resistance. The currently known aminoglycoside modifying enzymes utilize three common mechanisms: aminoglycoside phosphotransferase (APH), aminoglycoside acetyltransferase (AAC), and aminoglycoside nucleotide transferase (ANT).^249,250^ These enzymes trigger the resistance of different antibiotics through various mechanisms, providing powerful resistant activities towards different types of antibiotics. APH can inactivate streptomycin by transferring the phosphate group to the 3′-hydroxyl group of aminoglycosides.^251,252^ AAC may cause gentamicin resistance by transferring the acetyl group to the amino group at the 3′ and 6′ positions of the aminoglycoside.^253^ ANT confers *P. aeruginosa* resistance to amikacin by transferring adenosine groups to the amino or hydroxyl groups of these aminoglycosides.^254^ The diverse enzyme list is growing (currently more than 50 enzymes), which helps in bacterial success in the anti-drug battle with humans.

### Acquiring the antibiotic resistance throughout bacterial life cycle

Bacteria can acquire antibiotic resistance through mutations or horizontal gene transfer.^255,256^ Mutations of OprD in *P. aeruginosa* confers resistance to carbapenems^234^ and mutations of DNA gyrase (GyrA) causes resistance to quinolone antibiotics.^257^ Importantly, mutations in the β-lactamase gene *ampC* causes a significant increase in resistance to cephalosporins.^258^ There are already a host of enzymes in this bacterium may counter antibiotics while it continues to gain new resistance factors, which is debatably the biggest challenge for drug industry and scientific research. As bacteria can conveniently obtain antibiotic resistance genes from the same or different bacterial species through horizontal gene transfer, despite challenging targeting this mechanism may be a niche to search for better treatments.^259^ A typical example is that *P. aeruginosa* may obtain aminoglycoside and β-lactam resistance genes through horizontal gene transfer from the environment or other microbes at an unpredictable, fast pace,^223,260^ it may be highly difficult for scientists and clinicians to design new tools in impeding this natural robust mechanism in this bacterium.

### Adaptive antibiotic resistance

When facing the diverse environmental stimuli, bacteria obtain adaptive resistance to increase the resistance to antibiotics through transient changes in gene and/or protein expression by a spectrum of approaches.^261^ In *P. aeruginosa*, the formation of biofilms is the most typical strategy to acquire adaptive antibiotic resistance.^262,263^ *P. aeruginosa* can produce biofilms to enhance pathogenicity. Meanwhile, *P. aeruginosa* infection can also cope with antibiotic treatment by forming persistent cells or persisters, thereby preventing the synthesis of antibiotic targets.^223,262,263^ Persistent molecules from the persisters can maintain vitality and refill biofilms; once antibiotics are not present, bacteria will resume growth and cause chronic infections.^264^

Altogether, *P. aeruginosa* can become exceedingly resistant to antibiotics through myriads of mechanisms, and currently we probably only know a tip of iceberg regarding these bacterial behaviors. It necessitates accelerated research efforts to fully understand the detailed mechanisms by which bacteria constantly grow antibiotic resistance, providing insight into the design and development of innovative and efficious drugs to overcome drug resistance.

## Highly complex and multiple mechanisms in host immune responses to the bacterium

The opportunistic pathogen *P. aeruginosa* exists almost everywhere and in any environmental conditions. In immunosuppressed people, there is extreme susceptibility to *P. aeruginosa* infection, developing either acute or chronic phenotypes. As the first line of host defense systems, the innate immune system plays a vital role in battling with *P. aeruginosa* via multiple mechanisms, such as phagocytosis and inflammatory responses. Several types of host systems, such as pattern recognition receptors (PRRs), plasma membrane signals, intracellular enzymes, and secreted cytokines/chemokines participate in inflammatory response against the bacterial infections. Although a well-balanced inflammatory response is required for restraining *P. aeruginosa* invasion, overzealous inflammation is associated with rapid disease progression, tissue injury, and even death. Some host molecules including cytosolic protein annexin A2 (AnxA2), autophagy-related protein 7 (ATG7), NLRC4, as well as non-coding RNAs (lncRNAs and microRNAs), are also implicated in *P. aeruginosa*-induced inflammation and/or other aspects of host defense mechanisms,^265–267^ and understanding of the mechanisms of inflammatory response is just beginning to be unfolded.

### Toll-like receptors (TLRs) instantly recognize invasive pathogens

TLRs are highly conserved transmembrane PRRs, containing leucine-rich repeats and Toll/interleukin-1 receptor (TIR) homolog domains, which rapidly recognize invading microorganisms and elicit innate immunity and inflammatory response upon bacterial invasion.^268^ TLRs play vital roles in initiating innate immunity for eradicating invading pathogens.^269^ Upon the sensing of pathogen-associated molecular patterns (PAMPs), TLRs activate NF-κB and AP-1 to mediate inflammatory response signal pathways.^270^ Correspondingly, TLRs are capable of recognizing different pathogen-associated molecular patterns found in *P. aeruginosa*. LPS is a major component of the outer membrane of *P. aeruginosa*, responsible for maintaining bacterial membrane structure and initiating immune response as a major antigenic factor. Research shows that a large amount of hex-acylated lipid A from LPS is isolated from infected patients, which is a strong TLR4 agonist, capable of activating TLR4-dependent inflammatory response.^271,272^ In addition, several TLR4 co-receptors MD-2 and CD14 are indicated necessary for TLR4 recognition of LPS and TLR4 activation.^273^ Airway epithelial cells and macrophages, both expressed TLR4 in the cell membrane, serve as the first line of host defense for the initial contact to *P. aeruginosa*. As TLR4 is the essential trigger of host immunity against *P. aeruginosa* infection, its deficient mice show higher bacterial burdens and severe lung injury under infection.^274^ Nevertheless, the TLR4 pathway does not function alone and is very complex, which may be impacted or coregulated by a number of signaling systems including AnxA2,^275^ autophagy,^276,277^ inflammasome,^278^ cGAS,^279,280^ ion channel proteins,^281^ DNA repair proteins,^282^ miRNAs,^40,266,283^ and lncRNA,^229^ to name a few.

Apart from TLR4, TLR2 has also been reported as an LPS recognizer, which is capable of recognizing *P. aeruginosa*‐derived lipoproteins and T3SS effector ExoS.^269^ In addition, TLR5 is responsible for detecting *P. aeruginosa* flagellin, which can induce specific inflammasome.^284^ TLR9 is responsible for detecting *P. aeruginosa* unmethylated bacterial CpG DNA intracellularly.^285^ Interestingly, TLR9 is also activated by *P. aeruginosa* infection-induced mtDNA release (Wang Biao et al., under revision, Immunology).

Although paradoxical at times, TLRs are arguably the single most important PPRs for initiating the initial recognition to mount a robust immune defense against both bacteria and viruses. It is imperative that scientists be focused on verifying the roles of played out by TLRs using a comprehensive approach or systems biology in an unbiased manner in animals as well as human subjects,^277^ which may provide insight into the disease pathogenesis and suggesting new therapeutic development.

### Inflammasomes drive inflammation and pyroptosis

The inflammasome is a multiprotein complex, which is attributed to the production and release of inflammatory cytokines, IL-1β and IL-18.^286,287^ Recent work reveals that inflammasome is involved in pyroptosis dependent on the cleavage of Gasdermin D, which contributed to the formation of plasma membrane pores, and in turn promoting the release of inflammatory cytokines and pyroptosis.^288,289^ Typically, inflammasome consists of cytosolic PRRs, ASC (an apoptosis-associated speck-like protein containing a CARD), and caspase 1. Inflammasome PRRs are responsible for detecting exogenous PAMPs like TLRs, which are also essential for monitoring *P. aeruginosa* invasion and activate host inflammatory response for promoting the clearance of *P. aeruginosa.*^269,290^ Remarkably, TLRs are involved in the priming of inflammasome activation by promoting the transcription of inflammasome-related genes.^290^ NLRC4 and AIM2 (absent in melanoma 2) are well characterized among numerous inflammasome PRRs linked to infection of *P. aeruginosa*.

NLRC4 inflammasome is shown capable of recognizing needle and inner rod (PrgJ) T3SS proteins, independently of T3SS exotoxins, or intracellular flagellin utilizing different murine NAIP as adaptors.^291–294^ Unexpectively, only one factor, human NAIP (hNAIP), has been found, homologous to murine NAIP5, responsible for sensing *P. aeruginosa* T3SS inner-rod protein PscI and needle protein PscF.^295^ Apart from T3SS, the T4SS pilin is also capable of activating inflammasome independent of NLRC4 and ASC.^153^ *P. aeruginosa*-induced mitochondrial dysfunction also promotes the assembly and activation of NLRC4 via T3SS.^296^ Mitochondrial ROS and release of mitochondrial DNA are key to NLRC4 activation under *P. aeruginosa* infection, which is also dependent on autophagy.^296^ Removal of damaged mitochondria blocks the activation of NLRC4.^296^ However, AIM2 appears to be dispensable for recognizing and promoting *P. aeruginosa* infection-induced inflammation in most cases.^297^

Although several *P. aeruginosa* components are implicated in activating inflammasome-related immune defense, little is known about how the bacterium evades immune response after inflammasome activation.^298^ Recent research broadens our knowledge that *P. aeruginosa* takes advantage of bacterial QS-dependent secretant, including proteases, pyocyanin, and 3-oxo-C12-HSL, to inhibit the activation of NLRC4 or NLR family, pyrin domain containing 3 (NLRP3) inflammasomes.^298^ A further study supports that pyocyanin specifically inhibits activation of the NLRP3 inflammasome (but not NLRC4 and AIM2) through induced excessive oxidation,^299^ contrary to the positive role of oxidation in NLRP3 activation.^300^ Hence, the potential role of oxidation in *P. aeruginosa* infection and inflammasome activation requires further study. In addition, K^+^ efflux is necessary for the activation of inflammasome against *P. aeruginosa* infection.^301^

To date, the role and underlying mechanisms of the inflammasome in host defense against *P. aeruginosa* remain largely unclear and sometimes paradoxical: some researches show that inflammasome activation enhances host defense to clear *P. aeruginosa,*^302,303^ whereas others present opposite results that inflammasome activation triggered severe host tissue damage, which may impact disease progression and mortality.^304,305^ One explanation for such a mixed response is that infection may involve different pathways dependent on the bacterial strains, model systems, environments, and experimental conditions. Another caveat is that most experiments have not been performed at holistic and/or spatiotemporal levels to evaluate the dynamics, rather a single cell type, specific location, and one or two timepoints. Therefore, enhanced or advanced approaches are needed to further clarify the role and underlying regulatory mechanisms of inflammasome activation in *P. aeruginosa* infection.^277^

### Nucleic acid sensor cGAS is pivotal for eliciting immune reaction

cGAS is a recently discovered nucleic acid sensor that is initially identified to recognize viruses.^306^ Typically, activation of cGAS contributes to the induction of inflammasome as a means against viral or intracellular bacterial infection.^307^ However, the latest research shows that cGAS downstream effector STING may also play an anti-inflammatory role under extracellular bacterial *P. aeruginosa* infection by inhibiting NF-κB activity.^308^ More recently, we have found that cGAS may be involved in an uncoupled protein response (UPR) during *P. aeruginosa* infection. Mechanistically, our studies uncover cGAS as a novel nucleic acids’ sensor to initiate immune responses against *P. aeruginosa* infection through a canonical pathway involving STING and IRF-1,^280,309^ suggesting that cGAS pathways may be a critical target for control of this bacterium. It is a new and important function for cGAS since previous reports were primarily focused on viral sensing and intracellular bacterial sensing.

### CRISPR-mediated adaptive immunity, a double-edged sword for bacterial self-survival and invasion

**Fig. 5:**
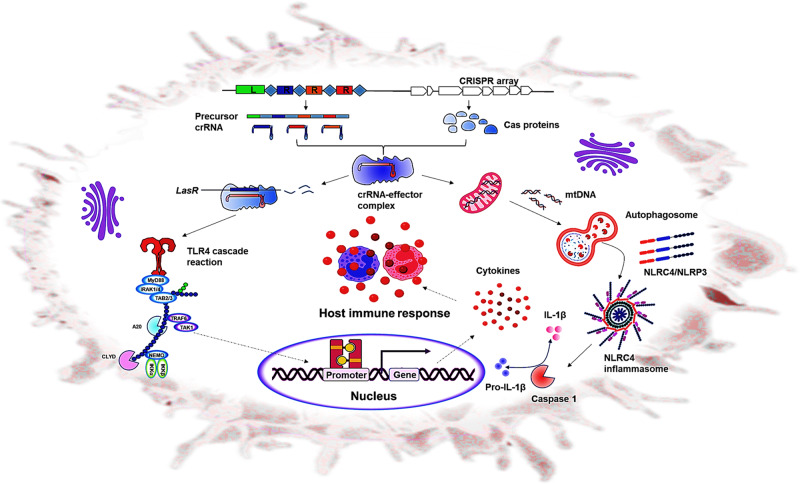
CRISPR-mediated adaptive immunity. Type I-C, type I-E, and type I-F CRISPR-Cas systems have been identified in *P. aeruginosa*. Type I CRISPR-Cas targeted endogenous *LasR* gene to decrease TLR4 expression and TLR4-mediated host inflammatory responses. Similarly, type I CRISPR-Cas systems elicited inflammasome activation by promoting mitochondrial-mediated autophagy. Ultimately, CRISPR-mediated adaptive immunity helps *P. aeruginosa* evade mammalian host immunity

CRISPR-Cas (clustered regularly interspaced short palindromic repeats-CRISPR-associated) is widely existing in bacteria and archaea providing protection against genetic intruders (plasmids), phages (phage) and other parasites.^310^ To date, two classes, six types of CRISPR-CRISPR-associated (Cas) systems, based on the characteristic of Cas proteins, are present in bacteria.^311–313^ Class I CRISPR-Cas systems (types II, V, and VI systems) rely on multiple CRISPR-Cas protein effector complexes, while Class II CRISPR-Cas systems (types I, III, and IV systems) are dependent on a single CRISPR-Cas effector protein.^314–316^ CRISPR-Cas I-C, type I-E and type I-F systems have been found in many clinically isolated *P. aeruginosa.*^317,318^ It is known that CRISPR-Cas systems provide adaptive immunity against the invasion of bacteriophages or plasmids.^319^ However, the role of CRISPR-Cas systems is far beyond the adaptive immunity of bacteria based on the current reports.^320^ Our research showed that type I CRISPR-Cas targeted the QS regulator *LasR* to inhibit toll-like receptor 4 recognition, thereby evading mammalian host immunity, suggesting that the CRISPR system is linked to host immunity modulation by targeting their own genes, potentially evading host defense mechanisms.^321^ Type I CRISPR-Cas systems may elicit inflammasome activation in mammalian hosts by regulating autophagy in *P. aeruginosa.*^278^ To ensure maximum CRISPR-Cas function, *P. aeruginosa* PA14 utilized quorum sensing to activate the expression of *cas* genes to promote CRISPR adaptation when bacteria are at high risk of phage invasion.^322^ The adaptability and virulence of *P. aeruginosa* can be regulated by CRISPR-Cas adaptive immunity based on the biological complexity of microbial communities in natural environments.^323^ The CRISPR-Cas system actively maintains the virulence of *P. aeruginosa* by limiting virus-like accessory genomic elements.^324^ Increased expression of phage-related genes initiates CRISPR-mediated biofilm-specific death of *P. aeruginosa.*^325^ CRISPR-Cas systems may help mediate antibiotic resistance to multiple membrane irritants, including enhancing the integrity of the envelope in pathogen *Francisella novicida.*^326^ Consequently, various corresponding anti-CRISPR (Acr) proteins have evolved by phages to inhibit bacterial CRISPR systems.^315,327–333^ Our previous research identified a series of type VI-A anti-CRISPRs (acrVIA1-7) genes that block the activities of Type VI-A CRISPR-Cas13a system,^333^ and designed Type III CRISPR endonuclease antivirals for coronaviruses (TEAR-CoV) as an experimental therapeutic to combat SARS-CoV-2 infection,^334^ suggesting that exploiting the mechanism of CRISPR-mediated adaptive immunity has great potential for treating bacterial and viral infections. However, it is not clear whether CRISPR-Cas systems can regulate antibiotic resistance in *P. aeruginosa*, which may be studied in future (Fig. 5).

## Development of novel therapeutics for *P. aeruginosa* is not up to the speed

**Fig. 6:**
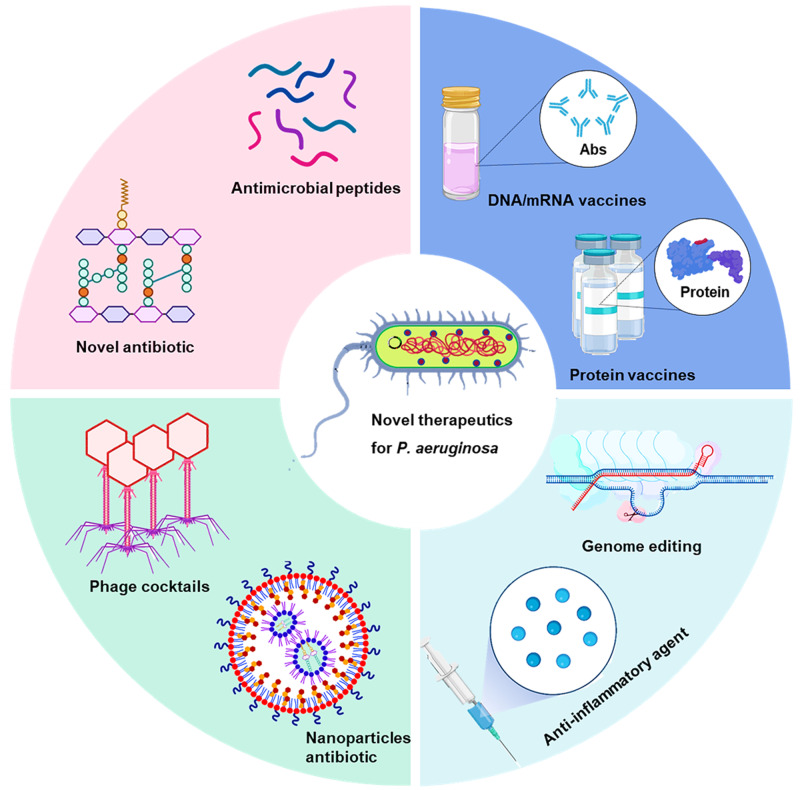
Novel therapeutics for *P. aeruginosa*. Multidrug-resistant *P. aeruginosa* poses a major challenge to traditional antibiotics therapeutics, which have limited efficacy and cause serious side effects. Phage therapy, immunotherapy, gene editing therapy, antimicrobial peptides, and vaccine therapies have become the most promising strategy and garnered great expectations to overcome multidrug-resistant bacterial infections. A full-scale network of regulatory understanding of *P. aeruginosa* virulence is expected to be unveiled, thus, we will be in a much better position for rationale drug design to control *Pseudomonas aeruginosa* infections

*P. aeruginosa* have strong ability to develop natural and acquired drug resistance through various mechanisms, including the production of antibiotic inactivating enzymes or antibiotic modifying enzymes, inhibiting the penetration of the drug through the cell wall, changing the target of antibacterial action, and limiting the drug to reach its target and adaptation to the adverse environment.^223^ Due to the increasing difficulty in treating antibiotic resistance strain infection, the development of the anti-*P. aeruginosa* therapy depends on targeting the resistance mechanism. Research on the resistance mechanism of *P. aeruginosa* has been an urgent topic for decades since antibiotic resistance has escalated exceedingly. Even with the intense interest, development of new antibiotics and other therapeutic strategies for *P. aeruginosa* infections is at a painstakingly slow pace due to the variability and complexity of drug resistance, as well as the lack of a deep understanding of the pathogenic mechanisms for *P. aeruginosa*. Designing effective therapeutic approaches (including phage therapy, immunotherapy, gene editing therapy, antimicrobial peptides, and vaccine therapies) to counteracting *P. aeruginosa* invasion has been an increasing urgency, requiring consorted efforts (Fig. 6).

### New antibiotic formulations and compounds

With an alarming rise in pathogens with resistance to existing drugs, a number of new antibiotics have recently entered the antibiotic development pipeline; however, the hope for patients and clinicians is rapidly dwindling once a new antibiotic resistance strain emerges. Hence, we should invest unconventionally research efforts in searching for new treatments of MDR bacteria. Recently developed antimicrobials are discussed below.

The biological activity of substituted guanidines was known in the mid-1930s when a series of guanidines and metformin compounds were found to possess bactericidal and disinfectant properties. Subsequently, many guanidine derivatives were studied for therapeutic purposes. Chin et al., recently reported a polyguanidine polycarbonates with strong antimicrobial activities through a distinctive mechanism that does not intensify drug resistance in multi-drug resistance (MDR) superbugs including methicillin-resistant *S. aureus, P. aeruginosa, A. baumannii, K. pneumoniae,*^335^ suggesting that the polyguanidine compounds may be potential antibacterial candidates.

In addition, novel antimicrobial peptides are promising to become the next generation of antimicrobials. The sequences of amino acids with antibiotic nature can be found in insects, soil bacteria, amphibians, plants, and even mammals. With the diversity of species and antimicrobial mechanisms, peptides render a niche as an alternative treatment for the MDR bacteria.^336^ A variety of new antibiotic formulations for treating *P. aeruginosa* infection have been tested in the clinic despite mostly at preliminary stages. Plazomicin is a novel semi-synthetic parenteral aminoglycoside that inhibits bacterial protein synthesis, and is shown to inhibit *P. aeruginosa* growth.^337^ Plazomicin is assessed in two Phase III randomized controlled trials, with an EPIC trial compared with meropenem in Complicated Urinary Tract Infection (cUTI). Plazomicin demonstrated a composite cure 81.7% (P) vs. 70.1% (M), a difference of 11.6%.^337^ Similarly, another trial CARE with plazomicin-based or colistin-based combinations to treat infection by carbapenem-resistant Enterobacteriaceae (CRE) shows that therapy with plazomicin-based combinations reduced mortality and complications vs. colistin-based combinations (23.5% vs 50%). This latter (CARE) trial seems more effective than the former (Plazomicin) and is achieved by enhanced sustained microbiological eradication.

Eravacycline is a novel fluorocycline that is evaluated for antimicrobial activity against anaerobic Gram-negative and Gram-positive bacteria. Eravacycline demonstrates potent broad-spectrum activity against 90% of bacterial isolates having a concentration range of ≤0.008 to 2 μg/ml.^338^ Eravacycline is more effective when an expression of tetracycline-specific efflux and ribosomal protection mechanisms is present. Eravacycline is effective against multi-drug-resistant bacteria towards other antibiotic classes. Eravacycline has shown broad-spectrum antibacterial activity when put unique chemical modifications at C-9 and C-7 of the tetracycline core.^338^

Baxdela, also known as delafloxacin meglumine, is a broad-spectrum anionic fluoroquinolone and has distinct chemical structures that increase potency in acidic environments. Delafloxacin is known for inhibiting DNA replication and repairs targeting DNA gyrase and topoisomerase IV. Currently, Baxdela is under a Phase III clinical trial for therapy of community-acquired pneumonia.^308^ Zidebactam (WCK 5222) is a dual mechanism antibiotic in a development phase, which is involved in binding Gram-negative PBP2 to exert β-lactamase inhibition. As an active antibiotic against Enterobacteriacease producing CTX-M-15, Zidebactam has some effects against Enterobacteriaceae and *P. aeruginosa.*^339^ Although we only reviewed a small portion of ongoing development of new therapeutics for *P. aeruginosa* with some hope, development of effective therapeutics to counter the growing drug resistance is still challenging.

### Nanoparticles may be useful for delivering drugs or vaccines

Nanoparticles have being tested in a number of therapeutic applications, such as drug delivery, gene delivery, and vaccine delivery in addition to direct killing of bacteria by its membrane piercing ability and induction of ROS. Since the CRISPR/Cas system can target to self-genes within a bacterium, delivery of CRISPR-Cas targeting a vital gene (for survival) by nanoparticles may directly kill the bacterium. Use of nanoparticles for delivering vaccines can also be achieved but preparation of optimal particles that can readily control protein loading and folding remains challenging.^340^ We will focus on the studies of vaccines and nanoparticles. There are several types of nanoparticles: nanoantibiotics liposomes, polymers, hydrogels, metallic particles, magnetic structures, carbon-based materials, and mesoporous with silica and each has its advantages and disadvantages. Hence, a multivalent nanoparticle strategy is considered a superior means and is used in vaccine delivery with *P. aeruginosa*, specifically to target T6SS. T6SS can divide into two distinct forms: phage tail-like structure and transmembrane complex, which can be exploited to create multivalent nanoparticle delivery of vaccine antigens. For the delivery of T6SS structural device, many proteins in a single nanoparticle may be delivered simultaneously.

The drawback of using nanoparticle structures is the likelihood that host immune responses may be activated against the delivery system, which can prohibit the repeated delivery.^340^ Future experiments need to be conducted to control antigen exposure to generate a uniform particle. The advantages of T6SS delivery system can be adopted for other gene targets with different nanoparticles. The advancement of nanotechnology has allowed a variety of nanostructures as a strategy to minimize the undesirable characteristic and natural and synthetic AMPs. Reports have indicated that in nanostructures, peptides present lower cytotoxicity and better efficiency of desired targets. Hence, nanostructures may help produce biomolecules and implementation of vaccines and drugs against extracellular degradation and target treatment. Using two approaches for encapsulating AMPs, nanotechnology can use an indirect approach where a passive delivery occurs involving a conventional nano-delivery system. Direct delivery is involved in an active target for modifying the surface chemistry of the nano-carrier with ligands. When comparing the two approaches, both have their rpos and cons, using the passive system involving the encapsulated peptide results in the best fitting solution.^336^

As biofilms are a major cause of drug resistance, nanoparticles targeting biofilms are also a promising strategy. Zhang et al. found that magnetite hybrid nanocomplexes can penetrate and disrupt bacterial biofilms by breaking through the biofilm matrix barrier.^341^ Others also demonstrate that silver nanoparticles (metallic particles) combined with polymyxin B can target *P. aeruginosa* biofilms with significant effects.^342^ Biofilms are also an important factor to aggravate cystic fibrosis disease. Hence, exotoxin A (ETA) is encapsulated into poly-lactic-co-glycolic acid (PLGA) nanoparticles, forming the ETA-PLGA nanoparticles, which may function as a continuous immunogen to trigger cellular immunity.^343^ Therefore, these nanoparticles may be one of the potential vaccine candidates to penetrate biofilms. To date, many nanoparticles-mediated compounds are in clinical development and several of them have been approved for human therapy, especially cancer therapy, hepatic fibrosis, virus infection, fungal infections, hypercholesterolemia, and pneumonia.^344–346^ In particular, the recently approved lipid nanoparticle (LNP) mRNA vaccines for COVID-19 was an acclaimed success in nanoparticle research for contribution to novel vaccines to combat diseases.^347^

### Phage therapy is a reviving alternative therapy for severe and incurable cases

Due to the lack of effective therapy for drug-resistant superbugs, phage therapy has been a viable alternative for bacterial treatment, especially for patients who have been non-responsive to conventional antibiotics. There are two types of bacteriophages, lytic and temperate, only lytic phages are utilized for clinical therapy. Lytic phages adhere to the host cell surface and inject DNA into host cells. Progeny phages release and migrate towards infection sites through targeting specific bacteria. For *P. aeruginosa*, flagella vaccines have been used together with phage or other therapies and showed the remarkable therapeutic results. However, *P. aeruginosa* displays heterogeneity and varying clinical outcomes with acute infections in later phases, where bacteria start losing their flagella and pili. A vaccine based on flagella will direct toward motile bacteria instead of established/colonized biofilms-forming strains.^348^ Advanced techniques have been developed for formulating phage cocktails to improve efficacy in vitro. For example, Forti et al. made a cocktail with six phages*: E215, E217, PAK_P1, PYO2, DEV,* and *PAK_P4* and showed some effects against clinical *P. aeruginosa* strains.^349^ A number of studies showed that phage cocktails possess enhanced efficiency in killing *P. aeruginosa* compared to single phage therapy.^350,351^ As strains from patients are distinct, personalized phages combined with antibiotics have also been applied for effective and safe therapy in clinics. An excellent such application was reported by Forti et al., who constructed phage cocktails based on the genomic information and host range.^349^ Clinical trials of phage therapy have shown viable and sustained efficacy in therapy. Marked reduction in bacterial colonies with a single dose of phage therapy has been reported in small studies.^352^ Previous studies have primarily been on patients with burns. Phages were applied on the wounds, but the minimal effect was observed.^353^ With *P. aeruginosa* being a ubiquitous pathogen that infects lungs and many other organs, further research will investigate how phage therapy would help control chronic CF or COPD diseases. Phage neutralizing antibodies that are induced by the body’s immune system and the safety concern about exotoxins during phage preparations are among the biggest challenge of phage therapy.

Another limitation is that bacteria can become resistant rapidly to phage therapy due in part to CRISPR-Cas systems, which are a bacterial adaptive immunity system that can degrade invading DNA in a high specificity. Research has been conducted with a CRISPR-Cas system that targets endogenous genes to alter host defense.^324^ To conquer the bacterial immune response for promoting phage replication, phages should gain access to do the job by evolving an anti-CRISPR system (Acrs). With this mechanism, future goals are to use natural anti-CRISPR or synthetic proteins that can counteract the CRISPR systems.^319^ A protein called AcrIF was shown to be a potent inhibitor of the CRISPR system in *P. aeruginosa*. This protein is locked with the Cys complex of CRISPR and diminished the phages’ ability to bind to DNA.^354^ Further research may be conducted using the same mechanism for bacteria to become resistant to CRISPR. Creating a synthetic phage with desired characteristics and genomic content would be a creative approach.^355^ Recently, mutations in phage host-range-determining regions (HRDRs) to cope with resistance mutation of bacteria have been implemented as an effective method to prevent *Escherichia coli* infection.^41^ Engineered bacteriophages BPsΔ33HTH_HRM10 and D29_HRMGD40 cocktail cured a cystic fibrosis patient with a disseminated drug-resistant *Mycobacterium abscessus*, which shows that the therapeutics of engineered phages to break the evolutionary constraints, holding great potential to create the next-generation of antibacterial antimicrobials.^356,357^ In spite of recent progress, the strategy for phage therapy is highly difficult. Through creative design, basic research and clinical testing, phage therapy can be rejuvenated against bacterial infections. Hence, it is promising to combine the phage therapy and anti-CRISPR approach for development of clinically feasible antibacterials.^333^

### Gene delivery has made notable progress

Genome editing and nucleic acid-based antibiotics, such as single-stranded DNA (ssDNA), double-stranded DNA (dsDNA), plasmid DNA, and ssRNA^358^ have emerged as the new types of antimicrobials. For gene editing, there is no doubt that editing with CRISPR systems is of high promise. Since scientists first used Cas9 to edit genomes in mice and human cells in 2013,^359^ the technology has been thought to have the greatest potential to cure some of the deadly diseases that humans cannot control today, including against bacteria-plagued diseases (i.e., CF), cancer and more importantly hereditary disorders. Although there is no therapy in clinics yet, the advance in off-target control and improved editing efficiency will translate gene therapy from bench to bedside in the foreseeable future. Both the gene editing and nucleic acid-based antibiotics need to be delivered into the host and cross spontaneously bacterial cell walls and membranes.

The major strategies for gene delivery are viral and non-viral vector systems. Non-viral strategy includes electroporation (direct injection, in vitro), lipids, and polymers. Research has also shown that LPN (Liposome polymer nucleic acid) is a versatile platform for efficiently delivering diverse nucleic acids to Gram-negative bacteria.^358^ Intracellular delivery of LPN can be directed against essential genes, resulting in bacterial growth inhibition or death.^360^ For viral delivery strategy, lentivirus, adenovirus-associated virus (AAV), and adenovirus vectors are often used.^361^ The lentiviral vector can efficiently integrate a foreign gene into the host chromosome, thereby achieving the effect of persistently expressing the sequence of interest. For some cells that are difficult to transfect, such as primary cells, stem cells, undifferentiated cells, *etc*., the use of lentiviral vectors has shown versatility to greatly improve the transduction efficiency of the target gene or the target shRNA. Therefore, in vitro and in vivo experiments, lentivirus has become one of the commonly used forms of expression for expressing foreign genes or exogenous shRNAs, and is gaining increasing attention.^362^ AAV’s function mechanism is very similar to lentiviruses. Compared to the lentiviruses, AAV shows better safety, a wide range of host cells, and low immunogenicity, so it has become the most promising gene transfer vector, especially for the CRISPR system.^363,364^ The AAV-based gene delivery may bear high transduction efficiency for delivering nucleic acid-based antibiotics, leading to better therapeutic effects.

### Development of vaccines is painfully slow

Nip in the bud, vaccines have been a viable approach for preventing millions of people from suffering devastating pandemic infections. Vaccine formulations can be efficiently targeted by antigen-presenting cells to generate both humoral and cellular immune responses.

Only can a few of vaccines that have being tested pass the hurdles of clinical trials to inoculate human populations. A major component of the bacterium targeted by vaccines are LPS and OMP with high rates of variations and mutations, which present challenges in vaccine designs. Vaccines based on these components have shown effects against homologous strains, but no effect against different serotypes. Two strategies have been adopted with LPS-vaccines: the first was to use lipid droplets for carrying LPS components into the host; and the second was stripping the lipid portion off LPS to create serotype-specific protection, which is considered the best option.^365^ Proteins or genetic vaccines appear to be the best candidates to vaccinate for *P. aeruginosa*. Emerging strains of *P. aeruginosa* are a key model system for investigating T4P structure and function, which may provide insight into vaccine design. Research indicates a serious nosocomial pathogen with facile growth requirements or contribution to motility.^366^ Flagellin and flagella have high immunogenicity, therefore, they are potential targets for vaccination against *P. aeruginosa.*^367^ The use of flagella is not only for motility purpose, but also for assisting attachment to the respiratory epithelium and activating TLR5. *P. aeruginosa* flagellin is a major unit of flagellar filament that is required to target protective antibodies and virulence. Flagella vaccines seem to help target acute infections and provide substantial relief of disease by preventing bacterial invasions and spreads. *Klebsiella* surface O polysaccharides (OPS) are protective antigens against infection in animals. The development of combined *K. pneumoniae* and *P. aeruginosa* glycoconjugate vaccine of four common *Klebsiella* OPS types may improve the control of human infections. OPS is chemically associated with two flagellin types, FlaA and FlaB.^368^ Monoclonal antibodies as passive immunity have shown protection of susceptible individuals to delay or prevent initial infections, especially those with cystic fibrosis.^369^ Increasing vaccines targeting iron chelation, lectin inhibitors, QS inhibition, have been developed. Finally, OMVs are being tested in clinical settings either vaccines or therapeutic carriers.^58^ Taken together, despite the strong interest in research and urgent need in public health, vaccines against *P. aeruginosa* are still unavailable for clinical application.

## Future directions

In the past several decades, although human beings have witnessed great scientific advances in basic research of pathogenesis and host defense as well as preventative and therapeutic development with *P. aeruginosa*, our understanding of this bacterium is still limited, impeding the hunt for novel effective therapeutic in the clinical settings. There are a large number of questions requiring clarification. (1) How do drug-resistant invasive strains emerge in the context with abuse of antibiotics or normal evolution? (2) How many other virulence factors are not discovered and characterized but critical for bacterial pathogenesis and evolution, especially those with remarkable virulence? (3) How the elements of the bacterium trigger and how the bacterium evade the activation, phagocytosis, and clearance by the human immune system? (4) What are new mechanisms responsible for increasing resistance in antimicrobial therapy? (5) How to better identify new host factors for improved immunity against this bacterium? (6) Can the scientific community establish a systematic and all-inclusive guideline to facilitate the discovery and revelation of novel antimicrobials, immune modulators, and other disease-modifying therapeutics?

## Summary

**Fig. 7:**
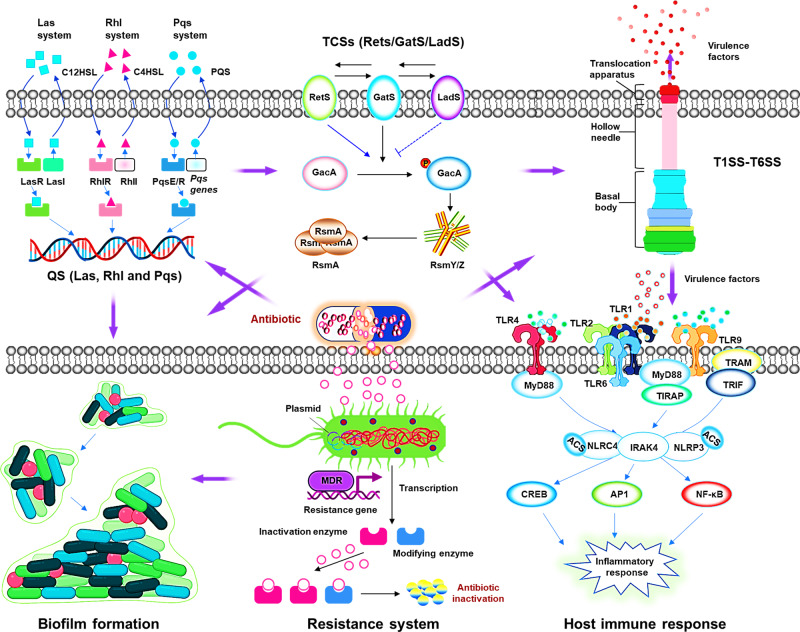
Interlinked and mechanistic regulatory network in *P. aeruginosa*. Interactions between various regulatory systems of *P. aeruginosa* are linked to regulate adaptation, survival, and resistance to multiple antibiotics, enabling *P. aeruginosa* to survive environmental stresses. QS systems primarily comprise Las, Rhl, and Pqs and are driven by autoinducer signaling molecules N-(3-oxododecanoyl)-L-homoserine lactone (3-oxo-C12-HSL) N-butanoyl-L-homoserine lactone (C4-HSL), and quinolone signal (PQS) activated through the interaction of transcription factors LasR/RhlR/Pqs and 3-oxo-C12-HSL/C4-HSL/PQS. When signaling molecules reach a putative threshold concentration in the cell environment, QS regulates toxicity expression by regulating two-component systems (TCSs). TCSs regulatory systems, consisting of sensor kinase and response regulator pairs, play roles in bacterial adaptation by regulating the expression of a variety of extracellular enzymes, virulence factors, and QS molecules. GacS/GacA TCS is regulated by sensor kinases RetS (positive regulation) and LadS (negative regulation). Transcription of non-coding regulatory RNAs of the RsmY/Z depends on the activation of GacS/GacA to activate RsmA and regulate the T3SS-mediated virulence secretion and biofilm formation. Biofilms are encapsulated in a self-generating extracellular polymer (EPS) matrix for species survival in surprising alterations of living conditions, like temperature fluctuations and nutrient availability, especially the antibiotic threat. Likewise, the secretory system activates the host immune response through virulence factors. TLRs play a key role in innate immunity. TLR1, 2, 4, 5, 6, and 9 are reported for recognizing *P. aeruginosa* infection and mediating inflammatory response signal pathways. T3SS and bacterial QS-dependent secretants have roles in modulating NLRP3 and NLRC4 inflammasome activation under *P. aeruginosa* challenge

As an opportunistic pathogen, *P. aeruginosa* has a complex regulatory system that is closely connected and mutually regulated to cope with the harsh external and internal environment, which causes substantial morbidity, debilitating diseases, shortened life span, and high mortality in humans (Fig. 7). In this comprehensive review and other articles, scientists have discussed the virulence factors of *P. aeruginosa*, such as LPS, adherence factors, elastase, secretion systems, and OMVs.^116,153,212,366,369–455^ We also introduced the recent progress in new antibiotic formulations and compounds, phage therapy strategy, vaccine approaches, nanoparticle fabrication as well as gene editing and nucleic acid-based antibiotics. Furthermore, we have included a large set of immune responses from hosts, including cell types, innate and adaptive immunity, and emerging advances in immunological research.

Our paper is a rarely extensive review that covers both bacterial pathogenesis and host defense in a great depth, serving as an irreplaceable reference for both students, doctors, and scientists who want to better understand *P. aeruginosa*. This comprehensive and analytic summary of current literature may enrich our knowledge in the balance between *P. aeruginosa* invasion and host responses. Despite the vast progress made over the years, a number of questions ranging from basic to clinical and applied aspects remain to be answered and further increased research efforts are still needed to study *P. aeruginosa*, which will improve our design to more effectively combat the infection caused by emerging drug-resistance strains.
